# Systems level profiling of chemotherapy-induced stress resolution in cancer cells reveals druggable trade-offs

**DOI:** 10.1073/pnas.2018229118

**Published:** 2021-04-21

**Authors:** Paula Saavedra-García, Monica Roman-Trufero, Hibah A. Al-Sadah, Kevin Blighe, Elena López-Jiménez, Marilena Christoforou, Lucy Penfold, Daria Capece, Xiaobei Xiong, Yirun Miao, Katarzyna Parzych, Valentina S. Caputo, Alexandros P. Siskos, Vesela Encheva, Zijing Liu, Denise Thiel, Martin F. Kaiser, Paolo Piazza, Aristeidis Chaidos, Anastasios Karadimitris, Guido Franzoso, Ambrosius P. Snijders, Hector C. Keun, Diego A. Oyarzún, Mauricio Barahona, Holger W. Auner

**Affiliations:** ^a^Cancer Cell Protein Metabolism, Department of Immunology and Inflammation, Imperial College London, London W12 0NN, United Kingdom;; ^b^The Hugh and Josseline Langmuir Centre for Myeloma Research, Imperial College London, London W12 0NN, United Kingdom;; ^c^Clinical Bioinformatics Research, London W1B 3HH, United Kingdom;; ^d^Cellular Stress, MRC London Institute of Medical Sciences, London W12 0NN, United Kingdom;; ^e^Centre for Molecular Immunology and Inflammation, Department of Immunology and Inflammation, Imperial College London, London W12 0NN, United Kingdom;; ^f^Department of Surgery and Cancer, Imperial College London, London W12 0NN, United Kingdom;; ^g^Proteomics Platform, The Francis Crick Institute, London NW1 1AT, United Kingdom;; ^h^Department of Mathematics, Imperial College London, London SW7 2AZ, United Kingdom;; ^i^Department of Brain Sciences, Imperial College London, London W12 0NN, United Kingdom;; ^j^UK Dementia Research Institute at Imperial College, London W12 0NN, United Kingdom;; ^k^Myeloma Molecular Therapy, The Institute of Cancer Research, Sutton SW7 3RP, United Kingdom;; ^l^Imperial BRC Genomics Facility, Department of Metabolism, Digestion and Reproduction, Imperial College London, London W12 0NN, United Kingdom;; ^m^School of Informatics, The University of Edinburgh, Edinburgh EH8 9AB, United Kingdom;; ^n^School of Biological Sciences, The University of Edinburgh, Edinburgh EH8 9AB, United Kingdom

**Keywords:** proteasome, myeloma, proteostasis, GCN2, metabolism

## Abstract

Cancer cells can survive chemotherapy-induced stress, but how they recover from it is not known. Using a temporal multiomics approach, we delineate the global mechanisms of proteotoxic stress resolution in multiple myeloma cells recovering from proteasome inhibition. Our observations define layered and protracted programs for stress resolution that encompass extensive changes across the transcriptome, proteome, and metabolome. Cellular recovery from proteasome inhibition involved protracted and dynamic changes of glucose and lipid metabolism and suppression of mitochondrial function. We demonstrate that recovering cells are more vulnerable to specific insults than acutely stressed cells and identify the general control nonderepressable 2 (GCN2)-driven cellular response to amino acid scarcity as a key recovery-associated vulnerability. Using a transcriptome analysis pipeline, we further show that GCN2 is also a stress-independent bona fide target in transcriptional signature-defined subsets of solid cancers that share molecular characteristics. Thus, identifying cellular trade-offs tied to the resolution of chemotherapy-induced stress in tumor cells may reveal new therapeutic targets and routes for cancer therapy optimization.

## Significance

Cancer therapies often fail to cure patients because a proportion of tumor cells withstand the toxic effects of chemotherapy. How surviving cancer cells recover from sublethal drug-induced stress is not known, but given that cellular resources are finite, stress resolution may come at the expense of less essential systems. Here, we studied the global cellular events of stress buildup and resolution in the bone marrow cancer, multiple myeloma, after proteasome inhibition, a commonly used therapeutic approach. Using a temporal multiomics approach, we delineate the unexpectedly complex and protracted changes myeloma cells undergo during stress resolution and demonstrate that recovering cells are more vulnerable to specific insults than acutely stressed cells. Thus, the findings may provide avenues for optimizing cancer therapies.

One of the distinguishing characteristics of cancer cells is their ability to overcome barriers that would normally negatively impact their survival, growth, proliferation, or metastatic spread (1). Cancer cells overcome diverse challenges such as nutrient scarcity, mechanical stress, or immune attack through cellular adaptations that enhance traits that increase their fitness in a selective environment. For example, cancer cells in hypoxic tumor regions activate a gene-expression program that rewires cellular energy metabolism, allowing them to thrive in limiting conditions (2, 3). However, given that cellular resources are finite, promoting adaptive hallmarks in one context is likely to come at the expense of decreased fitness in other selective conditions. In evolutionary biology, such effects are known as trade-offs (4–7) and are mirrored in cancer cell biology when tumor-promoting genetic or phenotypic changes simultaneously confer a vulnerability on alternative cellular processes (8–11).

Anticancer therapies are often administered in temporally spaced doses that each kill a fraction of tumor cells by causing overwhelming cellular injuries, while other cells survive. In this scenario, a substantial proportion of the remaining tumor cells nonetheless suffer from drug-induced stress that they need to resolve to survive and proliferate. The redistribution of cellular resources that is required for stress resolution is likely to decrease cellular fitness to withstand alternative challenges. In short, vulnerabilities linked to cellular recovery from anticancer therapies represent trade-offs that may reveal therapeutic targets and offer new routes for enhancing drug synergies. However, how cancer cells manage to resolve therapy-induced stress is not known.

The bone marrow cancer, multiple myeloma (MM), and its treatment with proteasome inhibitors (PIs) represent a scenario in which therapy-induced cellular fitness trade-offs can modulate clinical responses. PIs are proteotypical proteostasis-targeting drugs that by disrupting the ubiquitin–proteasome system, which is responsible for the controlled degradation of most cellular proteins, kill tumor cells through an array of proteotoxic effects both upstream and downstream of the proteasome, such as accumulation of misfolded proteins and impaired amino acid recycling for protein synthesis (12–16). Treatment typically consists of weekly PI doses that each eliminate a fraction of MM cells by triggering overwhelming stress, while other tumor cells survive. Clinically, this means that most patients respond to PI treatment but also that curative elimination of all cancer cells cannot be achieved (17). Working toward understanding the stress–recovery paradigm and using an integrated systems-level approach to study cellular events, we show that the MM cell transcriptome, proteome, and metabolome undergo unexpectedly complex and protracted changes during the resolution of PI-induced stress. We conclusively demonstrate that recovering cells are more vulnerable to specific insults than acutely stressed cells and identify mitochondrial respiration and the cellular response to amino acid depletion as druggable recovery-associated vulnerabilities. Moreover, we demonstrate that general control nonderepressible 2 (GCN2), a kinase that governs the resolution of amino acid scarcity (18, 19), is a bona fide therapeutic target in transcriptional signature-defined subgroups of diverse cancers irrespective of PI treatment.

## Results

### Resolution of Proteasome Inhibitor-Induced Stress Entails Protracted System Perturbations.

**Fig. 1.**
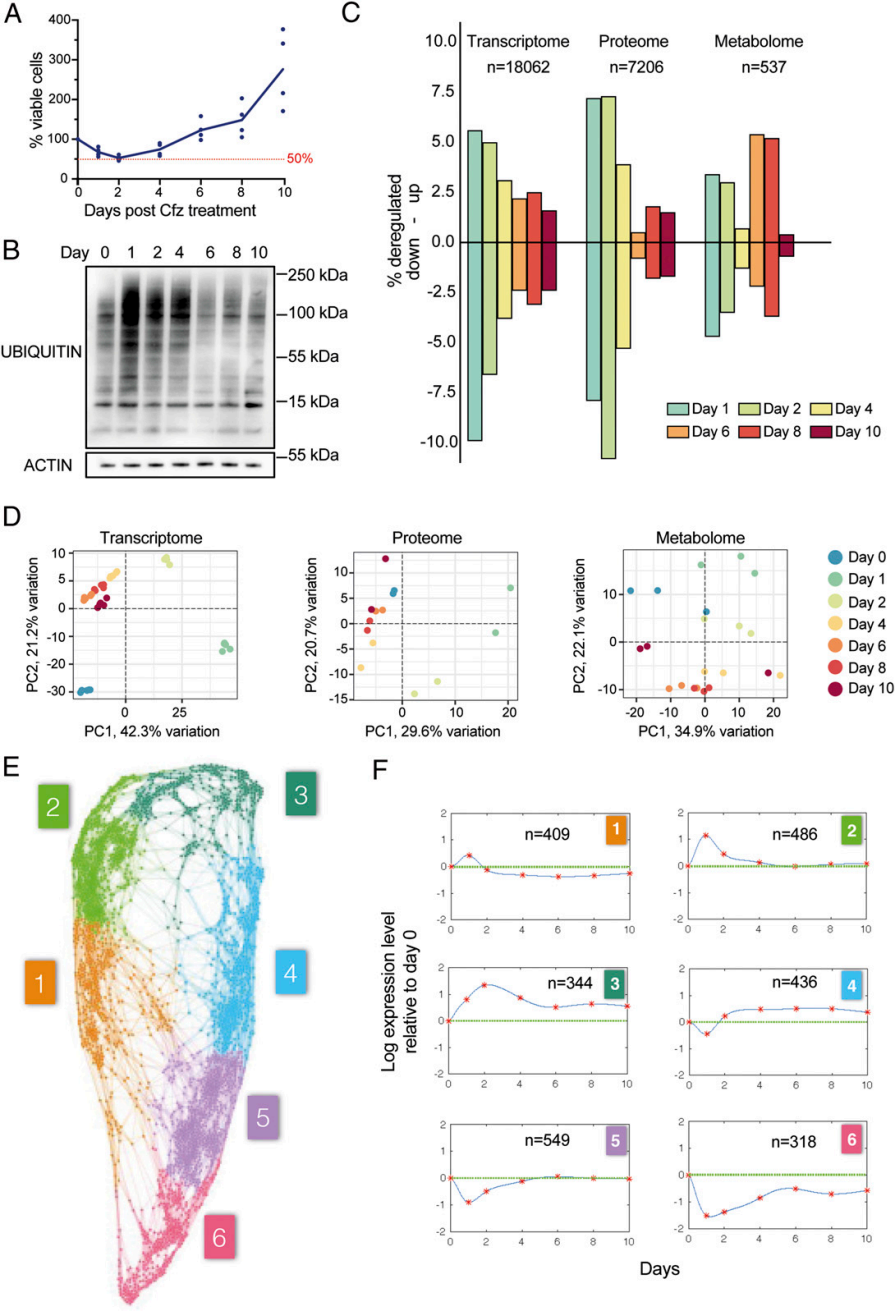
Global analysis of transcript, protein, and metabolite changes in MM cells recovering from proteasome inhibition. (*A*) Percentage of viable RPMI-8226 cells before and after carfilzomib (Cfz; 750 nM, 1 h) treatment (*n* = 4) as determined by Trypan Blue exclusion. (*B*) Immunoblot analysis of ubiquitinated proteins in whole-cell extracts from Cfz-treated RPMI-8226 cells (representative blot of *n* = 3). (*C*) Proportion of significantly deregulated transcripts, proteins, and metabolites (total numbers are indicated). (*D*) Unsupervised principal component (PC) analysis plots for transcripts, proteins, and metabolites. Percent (%) explained variation per PC is indicated by axis labels. (*E*) Gene-to-gene network for RNA sequencing data. Each node represents a transcript, and the connections between nodes represent the degree of similarity between their temporal response across all measured days; colors represent clusters of genes with similar temporal responses. (*F*) Average temporal expression profile of transcripts in clusters shown in *E*, also indicating the number of transcripts contained in each cluster.

To establish a clinically relevant in vitro model of PI stress recovery, we exposed RPMI-8226 MM cells to a 1 h pulse of the PI carfilzomib at 750 nM, which reduced the number of viable cells by ∼50% 2 d after the pulse (Fig. 1*A*). This approach closely replicates typical clinical pharmacokinetics and antitumor responses in MM patients (20, 21). We then carried out sequential transcriptome analyses by RNA sequencing, quantitative proteome analyses using a tandem mass tag (TMT) labeling approach, and metabolite profiling by liquid chromatography–mass spectrometry (LC–MS) at baseline and 1, 2, 4, 6, 8, and 10 d after treatment. At the same time points, we also collected mRNA and whole-cell protein extracts for quantitative real-time PCR and immunoblotting analyses, respectively, and cell culture supernatants for biochemical profiling by NMR spectroscopy. The number of viable cells reached a nadir on day 2 after proteasome inhibition and recovered to pretreatment levels on day 6 (Fig. 1*A* and *SI Appendix*, Fig. S1*A*). The amount of ubiquitinated proteins in whole-cell extracts as a readout of proteasome inhibition peaked on day 1 and then decreased to, or even slightly beyond, pretreatment levels on day 6 (Fig. 1*B*). A largely comparable temporal pattern of changes in viable cell numbers and ubiquitinated protein levels was observed in four other MM cell lines (*SI Appendix*, Fig. S1 *B* and *C*). Analysis of apoptosis and cell cycle in RPMI-8226 cells showed that the proportion of apoptotic cells peaked on day 4, while proliferation was lowest on day 2 and began to increase by day 4 (*SI Appendix*, Fig. S1 *D*–*F*).

To identify significant differences in transcripts, proteins, and metabolites compared to baseline (day 0), we used a 5% false discovery rate as cutoff, in line with comparable multiomics approaches (22, 23) (Fig. 1*C*). To enhance stringency further, a fold change > 2 was used as an additional cutoff for mRNA expression. The number of deregulated transcripts peaked on day 1 (*n* = 2,792 out of 18,062 transcripts, 15.5%) but was still at 4% (715 transcripts) on day 10. The highest number of deregulated proteins was observed on day 2 (*n* = 1,303 out of 7,206 proteins, 18.1%), while the largest proportion of deregulated metabolites was seen on day 8 (*n* = 48 out of 537 metabolites, 8.9%). We then performed principal component analysis (PCA) of transcriptomic, proteomic, and metabolomic data (Fig. 1*D* and *SI Appendix*, Fig. S1*G*), which showed separation of day 10 from day 0 samples, indicating that stress resolution was not complete on day 10. Moreover, PCA patterns indicated that the transcriptome, proteome, and metabolome of recovering cells differed from acutely stressed cells, suggesting that stress resolution was not a simple reversal of the processes that occurred during stress buildup.

In line with previous reports, we observed moderate but positive and significant correlations (r value range 0.222 to 0.344, *P* < 0.0001 for all days) between the fold changes of up- or down-regulated transcripts and proteins (22, 23) (*SI Appendix*, Fig. S1*H*). To further characterize the kinetics of the responses to proteasome inhibition, we focused on temporal changes in the transcriptome. First, using unsupervised machine learning applied to the time-course response of each transcript, we built a gene-to-gene network graph from the RNA-sequencing time series (Fig. 1*E*), where nodes represent transcripts, and the strength of connections between nodes represent the similarity of their time courses. Next, the network was clustered using a multiscale algorithm, which resulted in six clusters of 2,542 transcripts in total that represent the most prominent patterns of gene-expression changes (*SI Appendix*, Fig. S2*A*). Each cluster contained between 318 (cluster 6) and 549 (cluster 5) transcripts (Table S1) and was characterized by a unique temporal gene-expression profile (Fig. 1*F*). Pathway enrichment analysis in each cluster showed pronounced activity in pathways linked to the endoplasmic reticulum (ER) or proteasome-related protein processing, the unfolded protein response, and autophagy in clusters 1 and 2 (*SI Appendix*, Fig. S2 *B* and *C*), indicating that these processes were most active on day 1. Cell-cycle–associated pathway enrichment was predominantly found in cluster 3, indicating the reinitiation of proliferation during early recovery, in line with our cell-cycle analyses (*SI Appendix*, Fig. S1 *E* and *F*). In clusters 5 and 6, we observed enrichment of pathways related to protein processing at the ER and incorrect protein folding, and ribosome biogenesis and protein synthesis, indicating challenges of maintaining proteostasis while restoring a fully operational translational program.

### Proteasome Renewal and Oxidative Stress Dominate Early Proteasome Inhibition Effects.

**Fig. 2.**
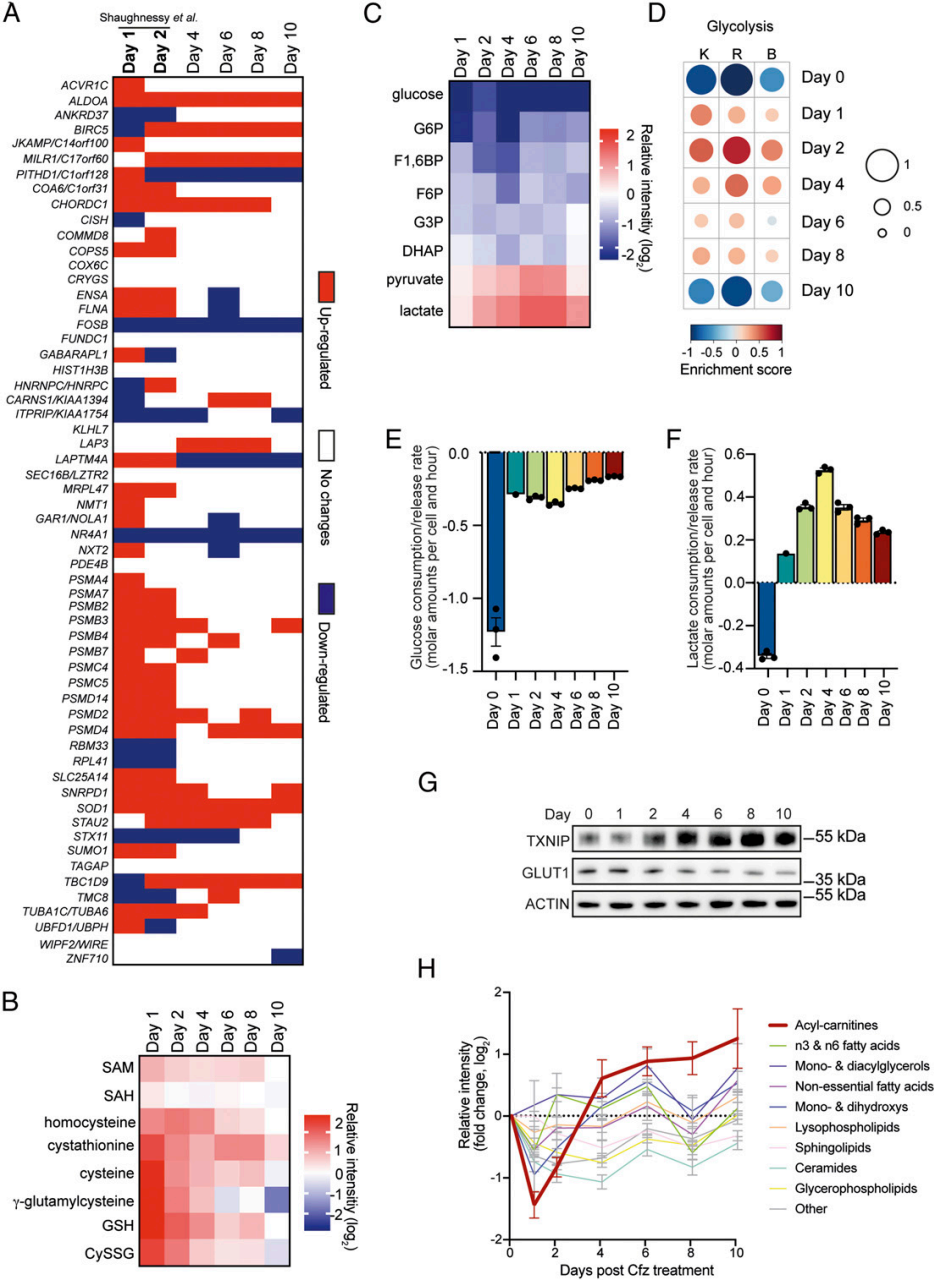
Recovery from proteasome inhibition entails oxidative stress resolution and triggers dynamic shifts in energy metabolism. (*A*) Heatmap showing deregulated and prognostic transcripts in myeloma cells derived from patients following treatment with a single dose of bortezomib (Shaughnessy et al.) (24) that were also identified by RNA sequencing in this study (red, up-regulated; blue, down-regulated; white, no significant change; two-way ANOVA, Dunnett’s test for multiple comparison, significance cutoff *P* < 0.05). (*B*) Heatmap representing relative levels of oxidative stress-related metabolites in carfilzomib-treated RPMI-8226 cells. SAM, S-adenosyl methionine; SAH, S-adenosyl-l-homocysteine; GSH, glutathione; CySSG, cysteine-glutathione disulfide (data shown as mean intensity, log_2_, of each metabolite normalized to day 0, *n* = 3). (*C*) Heatmap indicating relative levels of glycolytic metabolites. G6P, glucose 6-phosphate; F1,6BP, fructose 1,6-biphosphate; F6P, fructose 6-phosphate; G3P, glycerol 3-phosphate; DHAP, dihydroxyacetone phosphate (data shown as mean intensity, log_2_, of each metabolite normalized to day 0, *n* = 3). (*D*) Enrichment of glycolysis pathways as determined via GSVA of RNA-sequencing data. For visualization, the enrichment score range was scaled to be between −1 (underrepresented gene sets, blue) and +1 (overrepresented gene sets, red), also represented by the size of circles. K, KEGG; R, Reactome; B, Biocarta. (*E* and *F*) Metabolite consumption (negative values) and release (positive values) rates of glucose (*E*) and lactate (*F*) for RPMI-8226 cells based on NMR spectroscopy of cell culture supernatants (mean ± SEM, *n* = 3). (*G*) Immunoblot analysis of TXNIP and GLUT1 levels (representative blot of three independent experiments). (*H*) Changes in intracellular lipid-subfamily metabolites (mean ± SEM, *n* = 3) in response to carfilzomib (Cfz).

Focusing on events during stress buildup, we first compared our findings with those of the only study we are aware of in which the myeloma cell transcriptome of newly diagnosed patients was analyzed after a single in vivo dose of a PI (24). In this study, patients received a dose of bortezomib and underwent a bone marrow aspirate 48 h later followed by gene-expression profiling, which identified 65 genes with significantly altered expression that were highly survival discriminatory. Of those, 59 transcripts were captured by our RNA-sequencing approach. Despite differences in the gene-expression analysis platform and proteasome inhibitor used, and the heterogeneity of the patient population, 45 (76%) and 39 (66%) of the genes were also significantly deregulated on day 1 and 2, respectively, of our experiment (Fig. 2*A* and Table S2). These results highlight that our experimental model faithfully recapitulates clinically relevant effects of in vivo treatment with PIs.

One of the most striking early effects of proteasome inhibition we observed in line with the Shaughnessy study (24) was the quick and robust up-regulation of 35 proteasome subunit mRNAs, which were found predominantly in transcript cluster 2 (Table S1). This rapid but largely transient increase in transcripts in acutely stressed cells was observed for both 19S and 20S subunits. However, more 19S than 20S subunit transcripts subsequently dropped to below baseline levels during stress resolution (*SI Appendix*, Fig. S3*A*). Accordingly, gene set variation analysis (GSVA) also showed proteasomal pathway enrichment that peaked on day 1, followed by a return toward baseline (*SI Appendix*, Fig. S3*B*). Transcripts coding for the proteasome “bounce-back” regulator p97 (*VCP*) and its cofactors NPLOC4 and UFDL1 were also found in clusters 1 and 2. Thus, the cellular response by which proteasome subunits are renewed upon PI (25–27) was triggered rapidly.

Another prominent, early PI effect was the rapid onset of oxidative stress. Nuclear factor erythroid 2-related factor 2 (NRF2, encoded by *NFE2L2*) is a transcription factor that regulates genes, which contain antioxidant response elements in their promoters, and has been linked to PI treatment (28). We observed up-regulation of multiple NRF2 target-gene mRNAs predominantly in transcript cluster 2 (*SI Appendix*, Fig. S3*C*). Moreover, the NRF2 target HMOX1 (transcript cluster 2) stood out as the most highly expressed protein of all on day 1 (*SI Appendix*, Fig. S3*D*). We also observed a sharp and transient increase in levels of the major antioxidant, glutathione (GSH), its precursors, and its metabolite cysteine–glutathione disulphide (CySSG) (Fig. 2*B* and Table S3). Thus, a PI pulse rapidly triggered oxidative stress and a cellular response that gradually resolved it.

### Proteasome Inhibition Temporarily Enhances Glycolysis Followed by Increased Fatty Acid Catabolism.

Prompted by Kyoto Encyclopedia of Genes and Genomes (KEGG) pathway analysis of RNA-sequencing data that showed “metabolic pathways” as the most highly enriched term from day 2 to day 10 after PI treatment (*SI Appendix*, Fig. S3*E*), we investigated metabolic processes in more detail. One of the most striking metabolic changes was a profound and persistent decrease of intracellular glucose levels. While levels of glycolytic intermediates also dropped below baseline levels (Fig. 2*C* and Table S4), pyruvate and lactate progressively increased and reached peak levels on days 6 and 8, respectively, while the pyruvate/lactate ratio gradually decreased until day 10 (*SI Appendix*, Fig. S3*F*). GSVA of RNA-sequencing data showed that enrichment of glycolytic pathways, reflecting increased expression of glycolytic enzyme mRNAs, peaked on day 2 and then returned to baseline levels on day 10 (Fig. 2*D*). While these data suggested an increase in glycolytic activity during stress buildup and the early phases of stress resolution, analysis of corresponding cell culture supernatants by NMR spectroscopy showed that cellular glucose uptake decreased rapidly following proteasome inhibition and became even more suppressed during recovery (Fig. 2*E*). NMR data also showed that cells switched from lactate consumption at baseline to lactate release, which peaked on day 4 and then gradually decreased in recovering cells. (Fig. 2*F*). Looking for an explanation for the suppression of glucose uptake during recovery, we analyzed expression levels of the major glucose transporter, GLUT1, and of TXNIP, a suppressor of GLUT1 membrane expression and glucose uptake that is induced by high lactate levels (29–31). While GLUT1 levels decreased after day 2, TXNIP became increasingly up-regulated (Fig. 2*G* and *SI Appendix*, Fig. S3 *G* and *H*), providing a possible mechanistic link between increasing lactate levels and decreased glucose uptake during recovery. In addition to these complex changes in glucose metabolism, we also found that later stages of recovery were accompanied by increasing levels of acyl-carnitines and β-oxidation enzyme transcripts, suggesting enhanced β-oxidation (Fig. 2*H* and *SI Appendix*, Fig. S3*I*). Taken together, the results indicate that the cellular response to proteasome inhibition entails a dynamic shift in energy metabolism from increased glycolysis during acute stress to fatty acid catabolism during recovery.

### Proteasomal Stress Resolution Triggers Increased Mitochondrial Vulnerability.

**Fig. 3.**
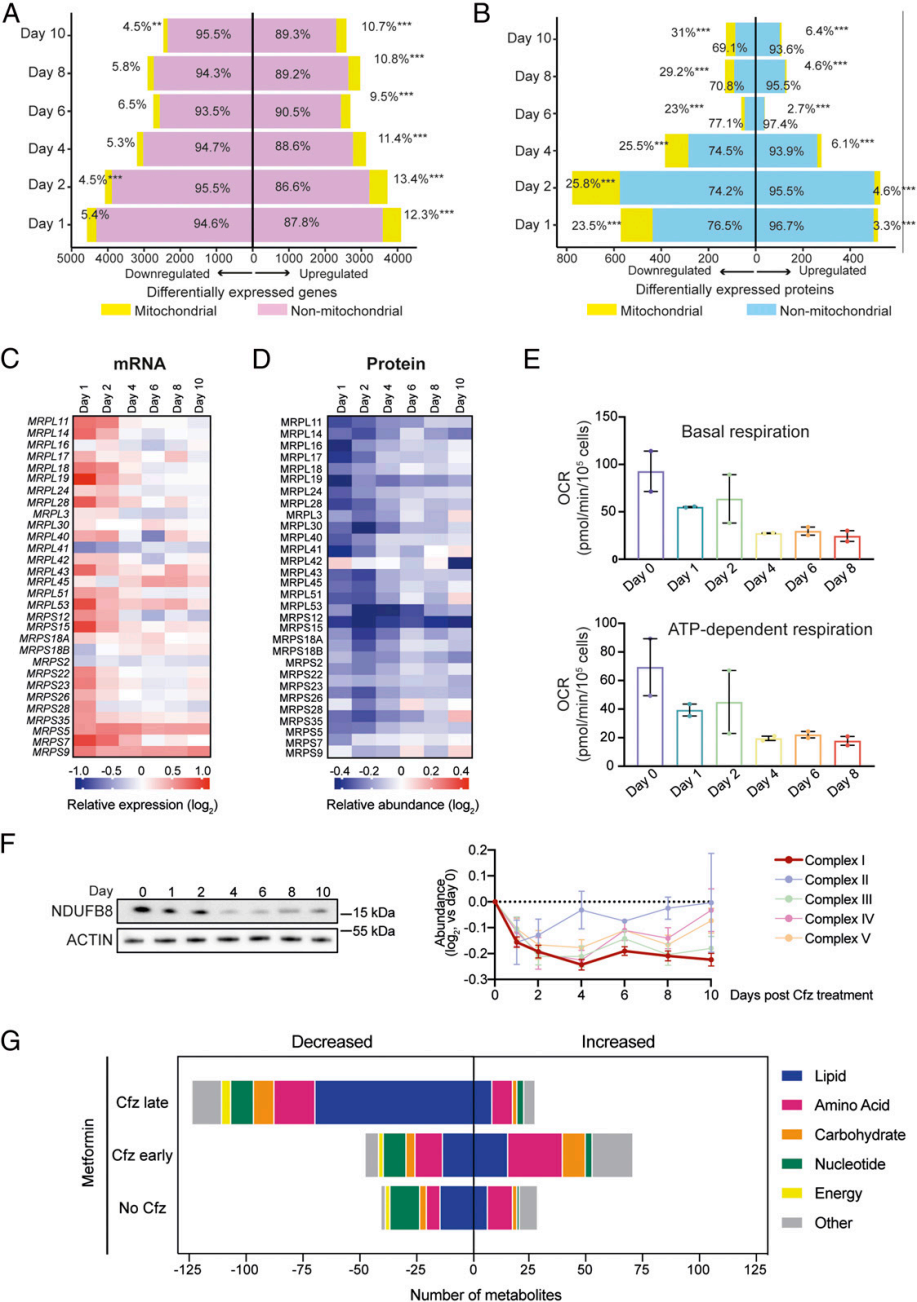
Mitochondrial changes during proteasome inhibitor-induced stress buildup and recovery. (*A* and *B*) Absolute numbers (x-axis) and proportion of significantly deregulated mitochondrial and nonmitochondrial genes (*A*) and proteins (*B*) based on MitoCarta2.0 presence or absence. Up- and down-regulated transcripts or proteins passing Benjamini–Hochberg *Q* ≤ 0.05 compared to day 0 were included. Level of statistical significance: ***, < 0.001; **, < 0.01; *, < 0.05. (*C*) Heatmap representing the expression levels of MRP transcripts (results shown as mean expression normalized to day 0, *n* = 5). (*D*) Relative abundance of TMT-labeled MRP peptides (data shown as mean levels normalized to day 0, *n* = 2). (*E*) Oxygen consumption rates (OCR) indicating basal (*Top*) and ATP-dependent (*Bottom*) respiration (mean ± SEM, *n* = 2 with 5 technical replicates each). (*F*, *Left*) Immunoblot analysis of NDUFB8 and actin (representative blot of *n* = 3); (*F*, *Right*) changes in ETC complex protein levels as determined by TMT-labeling analysis (mean ± SEM, *n* = 2). (*G*) Quantification of significantly altered metabolites in response to metformin as determined by LC–MS. Cfz-treated (day 0) RPMI-8226 cells were exposed to metformin (1 mM, 24 h) on day 0 (Cfz early) or day 5 (Cfz late; two-way ANOVA, 5% false discovery rate for multiple comparisons).

Increased glycolytic activity in cancer cells is often associated with a decrease in mitochondrial oxidative phosphorylation (OXPHOS). To determine how proteasome inhibition alters mitochondrial function, we first analyzed the mitochondrial transcriptome, using MitoCarta2.0 (32), and found that more genes encoding mitochondrial proteins were up-regulated than down-regulated from day 1 to 10 (Fig. 3*A*). GSVA of MitoCarta2.0 genes showed a biphasic signature enrichment that peaked on day 2 (*SI Appendix*, Fig. S4*A*), and we observed the highest proportion of MitoCarta2.0 genes in transcript cluster 2 (*SI Appendix*, Fig. S4*B*). In contrast, proteomic data revealed that significantly more mitochondrial proteins were down-regulated than up-regulated on days 1 to 10 (Fig. 3*B*). The discrepancy between transcripts and proteins prompted us to take a closer look at mitochondrial ribosomal proteins (MRPs), evolutionarily conserved and lifespan-regulating nodal points in mitochondrial stress communications (22, 33). We found that mRNAs coding for MRPs were largely up-regulated during stress buildup and returned to near-baseline levels during stress resolution (Fig. 3*C* and Table S5), in line with the predominant temporal pattern observed for MitoCarta2.0 genes. In contrast, protein levels initially dropped and then gradually increased to near-baseline levels during late stages of recovery (Fig. 3*D* and Table S6). These findings prompted us to test if mitochondrial respiration changed during stress buildup and recovery. Using Seahorse technology, we observed a reduction in the basal, maximal, and ATP-dependent oxygen consumption of viable cells on days 1 and 2, followed by a considerable further decrease throughout stress resolution (Fig. 3*E* and *SI Appendix*, Fig. S4*C*). Consistently, we found that electron transport chain (ETC) protein levels decreased during stress buildup and largely remained below baseline levels during recovery. Complex I proteins such as NDUFB8 were the most suppressed during recovery (Fig. 3*F*). This led us to test if mitochondrial stressors would have a different effect on recovering cells compared to acutely stressed or unstressed cells. We found that a panel of drugs that target ETC complexes, maintenance of the transmembrane proton gradient, or mitochondrial translation largely triggered a greater reduction in cell viability in recovering cells than in acutely stressed cells, although the effects only partly met statistical significance criteria (*SI Appendix*, Fig. S4*D*). Similarly, changes in transcript levels of ATF4, the main mitochondrial stress transducer (22), suggested that mitochondrial stress was enhanced by these agents at the same level or more in recovering cells compared to acutely stressed or unstressed cells (*SI Appendix*, Fig. S4*E*). We also determined the impact of metformin, a drug that exerts anticancer effects partly through OXPHOS disruption (34–36). While metformin was not overtly cytotoxic (*SI Appendix*, Fig. S4*F*), metabolite profiling showed that metformin perturbed a considerably larger fraction of the cellular metabolome when it was added to cell cultures during stress recovery compared to acutely stressed cells (Fig. 3*G* and Table S7). Moreover, the metabolic effects of metformin during stress resolution were profoundly different from the effects during stress buildup and were dominated by a significant decrease in the level of 70 lipidic metabolites, demonstrating that metformin perturbed the recovery-associated increase in fatty acid catabolism (*SI Appendix*, Fig. S4*G*). In contrast, ritonavir, syrosingopine, and GSK2837808A, drugs that target different metabolic processes (37–40), did not have a preferential effect on recovering cells (*SI Appendix*, Fig. S4 *H* and *I*), demonstrating that stress resolution does not result in universally increased vulnerability of the cellular metabolome. Taken together, these observations indicate that several aspects of mitochondrial function are compromised in cells recovering from PI stress and that these impairments may be tied to increased vulnerabilities to mitochondrial stressors.

### Intracellular Amino Acid Scarcity in Recovering Cells Triggers Dependency on GCN2 Signaling.

**Fig. 4.**
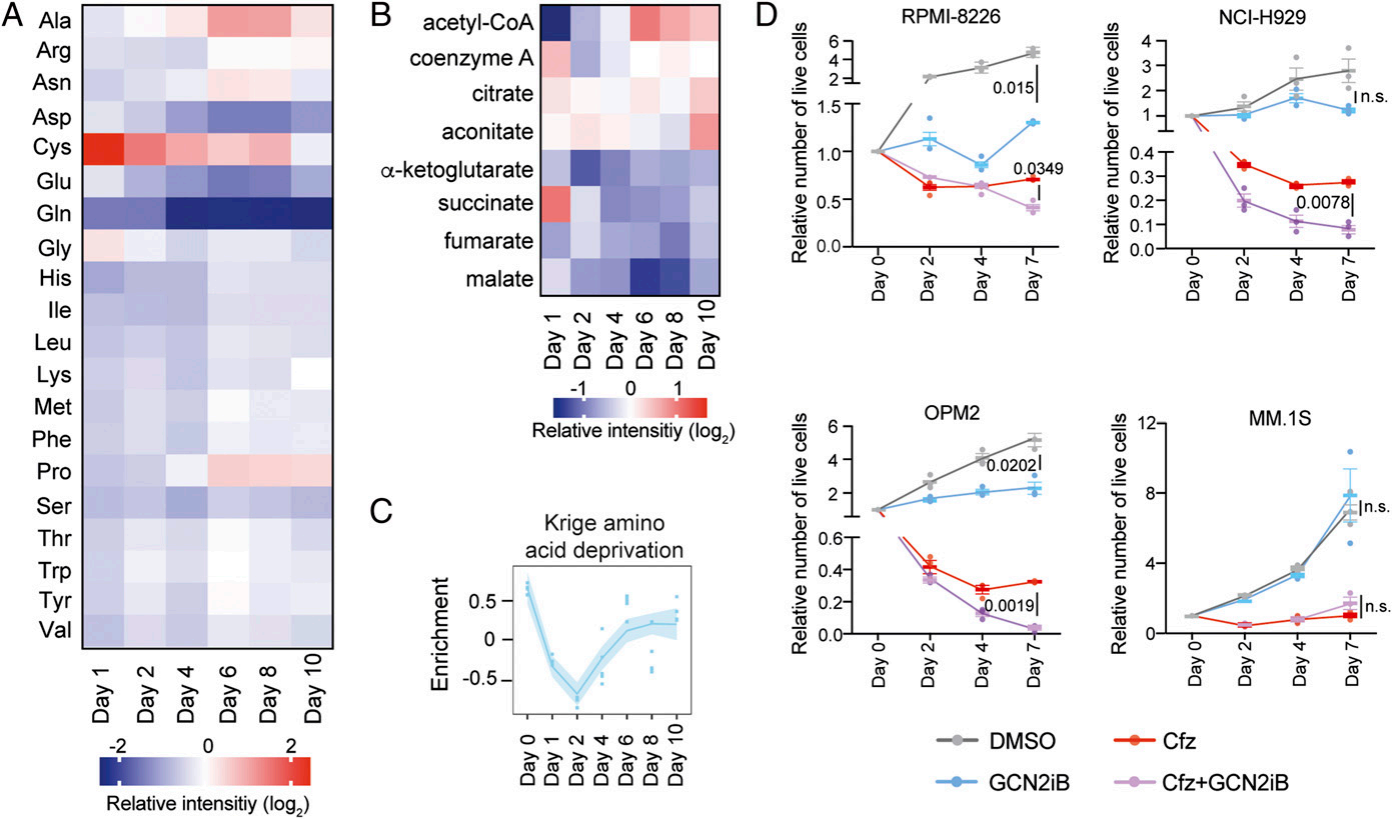
Proteasome inhibition causes amino acid depletion and GCN2 dependency during stress recovery. (*A*) Heatmap showing relative levels of amino acids in RPMI-8226 cells measured by LC–MS (data shown as mean intensity, log_2_, normalized to day 0, *n* = 3). (*B*) Heatmap depicting relative levels of TCA-cycle metabolites measured by LC–MS (data shown as mean intensity, log_2_, normalized to day 0, *n* = 3). (*C*) Krige amino acid deprivation–gene signature enrichment based on GSVA of RNA-sequencing data. (*D*) Effect of GCN2iB (1 μM, continuous for 7 d) on myeloma cell viability following a 1 h Cfz pulse (RPMI-8226, 750 nM; NCI-H929, 35 nM; OPM2, 100 nM; MM.1S, 75 nM). Viable cell numbers were determined by Trypan Blue exclusion (mean ± SEM, two-way ANOVA and Tukey’s test for multiple comparisons, *n* = 3).

Amino acids contribute to the generation of tricarboxylic acid (TCA)-cycle intermediates and thus the provision of reducing equivalents that drive OXPHOS (41). We found that different amino acids were altered in strikingly different ways during PI-induced stress buildup and recovery (Fig. 4*A* and Table S8). Cysteine levels increased rapidly but transiently, in line with an early oxidative stress response **(**Fig. 2*B*). Alanine levels increased from day 4 and peaked on days 6 and 8, which is compatible with increased pyruvate availability during recovery (Fig. 2*C*). However, levels of most amino acids decreased to below baseline in response to carfilzomib at some point. Most notably, glutamine levels dropped rapidly and remained low throughout recovery. Consistent with the established importance of glutamine anaplerosis in MM cells (42), and in line with the observed decrease in mitochondrial respiration, levels of glutamate and TCA-cycle metabolites α-ketoglutarate, succinate, fumarate, and malate also dropped to below baseline (Fig. 4*B* and Table S9). However, citrate and aconitate became more abundant during stress recovery, a finding that is compatible with the increased availability of acetyl-CoA downstream of pyruvate.

We then asked whether the observed reduction in amino-acid levels triggered a cellular response. When intracellular amino-acid abundance decreases, the ensuing increase in uncharged transfer RNAs (tRNAs) activates GCN2 (*EIF2AK4*) (18). Active GCN2 phosphorylates eIF2-α on serine 52, which triggers a largely ATF4-driven stress response often referred to as the integrated stress response (ISR) (19). First, we searched for key targets of the GCN2–ATF4 axis in the transcript clusters described in Fig. 1*E*. A group of genes encoding transmembrane amino acid transporters (*SLC7A11*, *SLC3A2*, *SLC1A5*, *SLC7A1*, and *SLC6A9*) were all found in transcript cluster 6 (Table S1). Moreover, genes encoding tRNA synthetases, enzymes that charge tRNAs with their cognate amino acids, were also largely found in clusters 5 and 6 (*AARS*, *CARS*, *GARS*, *MARS*, *QARS*, *SARS*, *TARS*, *WARS*, and *YARS*). Similarly, *EIF2AK4* and *ATF4* and functionally well-characterized major GCN2–ATF4 axis targets (*DDIT3*, *SESN2*, *ASNS*, and *CHAC1*) were also part of cluster 6. In contrast, mRNAs encoding key ER chaperones BIP (*HSPA5*) and P58IPK (*DNAJC3*), which are up-regulated by increased protein misfolding in the ER, and the ER stress transducer and eIF2-α kinase PERK (*EIF2AK3*), were not represented in any of the transcript clusters. GSVA of unfiltered RNA-sequencing data revealed a compatible enrichment pattern of an established amino acid depletion signature (KRIGE_AMINO_ACID_DEPRIVATION) and of ATF4 targets (IGARASHI_ATF4_TARGETS) (Fig. 4*C* and *SI Appendix*, Fig. S5*A*). Taken together, the findings reveal that, following a brief inactive period in the aftermath of proteasome inhibition, amino acid depletion-induced GCN2–ATF4 signaling becomes increasingly reactivated during recovery.

To ascertain if cellular recovery depends on a GCN2-driven stress response, we made use of the selective GCN2 inhibitor, GCN2iB (43). The choice of pharmacological inhibition over genetic depletion was driven by the requirement to rapidly switch off GCN2 signaling at precisely defined time points during stress buildup or recovery. First, we validated that GCN2iB disrupts stress signals triggered by amino-acid depletion but not by protein misfolding (*SI Appendix*, Fig. S5 *B* and *C*). Next, we tested if GCN2 inhibition affects myeloma cell growth. GCN2iB alone had a moderately inhibitory effect on the proliferation of RPMI-8226 cells. While GCN2iB did not enhance carfilzomib-induced cell death on days 2 and 4, GCN2 inhibition had a significant effect on viable cell numbers on day 7. When we extended our analysis to additional myeloma cell lines, we found that GCN2iB significantly enhanced the carfilzomib-induced reduction of viable OPM2 and NCI-H929 cells on day 7 but had no effect on MM.1S cells (Fig. 4*D*). Thus, GCN2 blockade disrupts cellular recovery from proteasome inhibition in a proportion of MM cell lines. Extending these data to nonmyeloma cells, and using a genetic targeting approach, we observed that short hairpin (sh)RNA-mediated depletion of GCN2 enhanced the cytotoxicity of proteasome inhibition in A549 lung adenocarcinoma cells (*SI Appendix*, Fig. S5*D*). We then tested the susceptibility of nonmalignant bone-marrow cells to GCN2 inhibition. To this end, we pooled primary mesenchymal bone-marrow stromal cells (MSCs) from three healthy pediatric MCS donors and exposed them to carfilzomib and GCN2iB. In line with previous observations using continuous bortezomib treatment (44), a 1 h carfilzomib pulse had a minor effect on the viability of MSCs, and inhibition of GCN2 did not result in any overt toxicity when given alone or following proteasome inhibition (*SI Appendix*, Fig. S5*E*). Taken together, the findings show that GCN2 promotes the resolution of PI-induced stress in cancer cells.

### GCN2 Blockade Highlights Its Intricate Metabolic Functions in Recovering Cells.

**Fig. 5.**
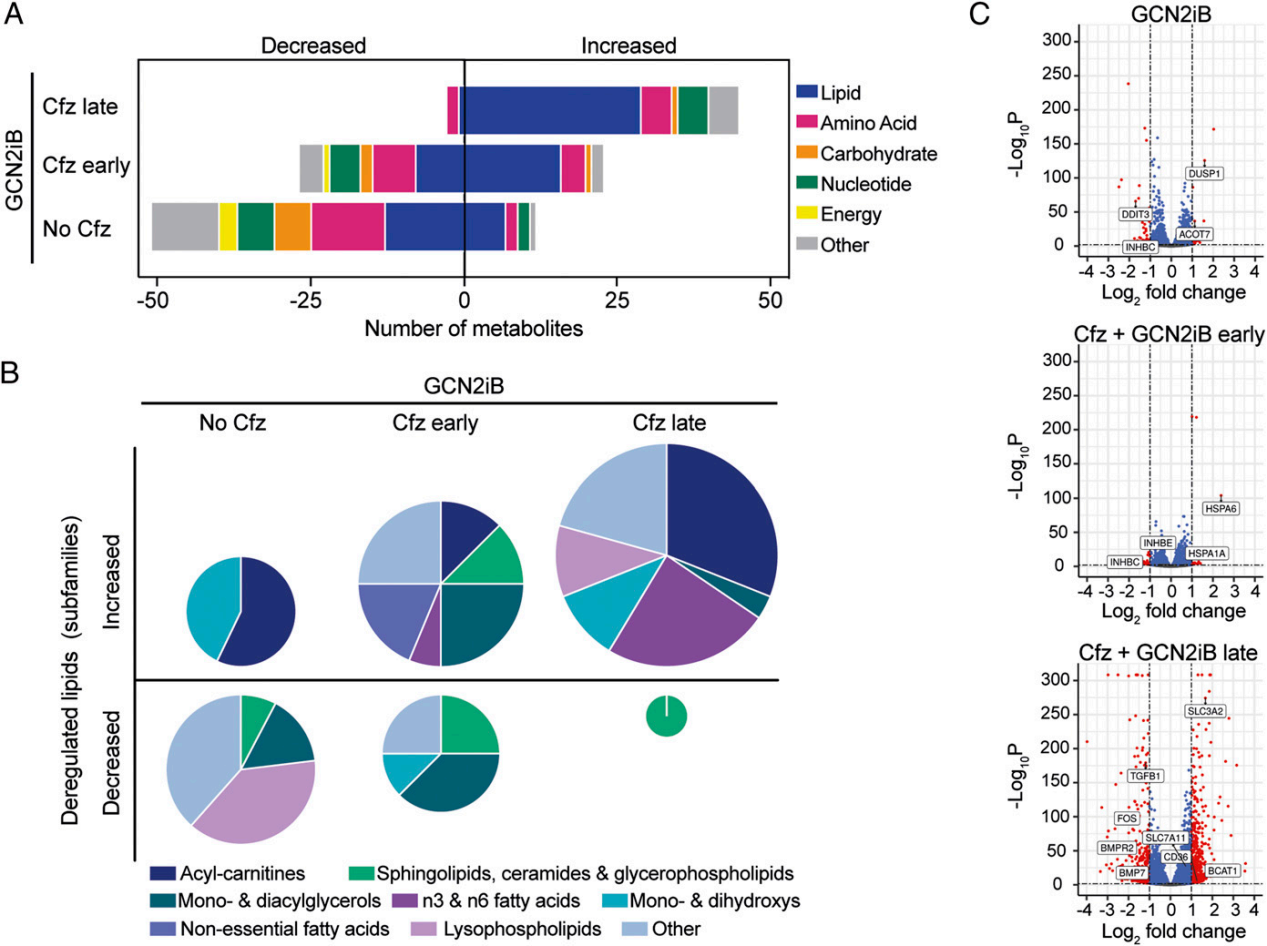
Differential effect of GCN2 inhibition on the cellular transcriptome and metabolome during stress recovery. (*A*) Quantification and classification of metabolites significantly deregulated by GCN2iB (1 μM, 24 h) in RPMI-8226 cells nontreated or treated with carfilzomib (Cfz; early, GCN2iB treatment on day 0; late, GCN2iB treatment on day 5) based on two-way ANOVA (5% false discovery rate for multiple comparisons). (*B*) Distribution of significantly deregulated lipids in lipidic subfamilies. Pie-chart sizes are representative of the number of deregulated lipids. (*C*) Volcano plots showing deregulated mRNAs after GCN2 inhibition. Cut-offs (dashed lines) are drawn at Benjamini–Hochberg *Q* ≤ 0.05 and absolute log_2_ fold change ≥1 based on RNA-sequencing–derived mRNA expression levels in RPMI-8226 cells treated with GCN2iB (1 μM, 48 h) early (day 2 to 4) or late (day 4 to 6) after a Cfz pulse, or without prior Cfz treatment. Selected genes of interest related to the ISR, TGF-β signaling, and fatty acid and cystine metabolism are indicated.

Next, to gain a mechanistic insight into the role of GCN2 in stress recovery, we determined the effects of GCN2 inhibition on the cellular metabolome. Biochemical profiling (Table S10) showed that pharmacological GCN2 blockade in unstressed cells predominantly triggered a decrease in the levels of diverse metabolites, including glutamine and aspartate, in line with the primary role of GCN2 in maintaining amino acid availability. However, GCN2 inhibition during PI-induced stress buildup had a qualitatively different effect, with lower levels of GSH, *N*-acetylcysteine, and cysteine pointing to a role for GCN2 in modulating oxidative stress. However, GCN2 inhibition during stress resolution triggered a different response. Out of 51 significantly altered metabolites, 45 were increased. Of those, 29 (64%) were classified as lipids, predominantly n-3 and n-6 fatty acids and acyl-carnitines, indicating a further metabolic shift toward β-oxidation (Fig. 5 *A* and *B*). Thus, the role of GCN2 in cells recovering from PI-induced stress is different from its function in acutely stressed or unstressed cells.

Next, we performed RNA sequencing to complement the biochemical profiling. We found that GCN2 inhibition significantly deregulated 95 transcripts in unstressed cells and that key GCN2–ATF4 targets with roles in amino acid homeostasis (*DDIT3*, *ATF3*, *CHAC1*, *SESN2*, *SLC7A11*, and *TRIB3*) were among the 71 down-regulated mRNAs (Fig. 5*C* and Table S11). When GCN2 was inhibited in cells that had reached the nadir in viable cell numbers (day 2 to 4), a lower number of mRNAs were deregulated than in unstressed cells, consistent with the lack of apparent cytotoxic synergy at this point. However, pharmacological GCN2 blockade in recovering cells (day 4 to 6) increased the number of deregulated transcripts more than 18-fold to 919 (down, 460; up, 459). GCN2 inhibition also increased 20S and particularly 19S proteasome-subunit transcript levels most during recovery, with a moderate effect in unstressed cells, and almost no detectable change in acutely stressed cells (*SI Appendix*, Fig. S6*A*). Focusing on the most relevant Gene Ontology (GO)-Biological Processes (BP) terms linked to GCN2 inhibition (*SI Appendix*, Fig. S6 *B* and *C*), we examined “Carboxylic acid metabolic process” in more detail and found that over 80% of up-regulated genes in this category code for proteins involved in amino acid or lipid metabolism, in line with metabolite data, while 36% of the down-regulated genes are involved in glucose metabolism (*SI Appendix*, Fig. S6*D*). We also found that components of the TGF-β pathway, which promotes MM growth and myeloma bone disease (45–47), were repressed by GCN2 inhibition during recovery (Fig. 5*C* and *SI Appendix*, Fig. S6*E*), including ligands (like *TGFB1* or *BMP7*), receptors (*BMPR2*), and effectors (*FOS*) (Table S11). RNA-sequencing results also showed that transcripts coding for both subunits of the cystine–glutamate antiporter (*SLC7A11* and *SLC3A2*) were up-regulated in GCN2iB-treated cells, in line with increased levels of cystine observed by LC–MS (Table S10). Together, the results demonstrate that cells that are recovering from proteasome inhibition have a heightened dependency on GCN2 to maintain homeostasis in multiple cellular systems.

### GCN2 Dependency Signatures in Cancer Subgroups.

Having identified GCN2 as a recovery-associated vulnerability in myeloma cells, we then set out to explore whether other cancer cells might be vulnerable to GCN2 irrespective of prior chemotherapy-induced stress. To this end, we made use of CRISPR essentiality screen data available in the Cancer Dependency Map (DepMap) (https://depmap.org/portal, CRISPR [Avana] Public 20Q1 release). In line with our finding that the cytotoxic effects of GCN2 inhibition on PI-naïve myeloma cell lines were absent or moderate, no myeloma cell line is classified as GCN2-dependent in DepMap. However, we found that 93 out of 739 cancer cell lines (13%) are dependent on GCN2 (*EIF2AK4*). By comparison, one, zero, and five cancer cell lines (0.1, 0, and 0.7%) are dependent on the other three eIF2-α kinases, *EIF2AK1* (HRI), *EIF2AK2* (PKR), and *EIF2AK3* (PERK). To test if gene-expression signatures can predict GCN2 dependency, we identified 61 cancer cell lines with the highest DepMap GCN2 dependency (median CERES scores −0.95; range −0.58 to −1.79) and 60 tissue-matched cell lines with the lowest GCN2 dependency (median CERES scores 0.21; range 0.0 to 0.61) (Table S12). Heatmap analysis of RNA-sequencing data revealed a clear difference in mRNA expression patterns between GCN2-dependent and GCN2-independent cell lines but was also indicative of tissue-specific heterogeneity (*SI Appendix*, Fig. S7). We therefore focused on skin cancer, the cancer type with the largest number of GCN2-dependent cell lines in DepMap. First, we identified a 56-gene signature that identifies GCN2-dependent skin cancer cell lines (*SI Appendix*, Fig. S8). We then projected this signature onto the transcriptomes of 424 melanomas in The Cancer Genome Atlas (TCGA) and found that 22 (5.2%) of the tumors matched the DepMap GCN2-dependency signature by more than 80% (*SI Appendix*, Fig. S9*A*). Heatmap analysis showed that these transcriptomes were distinct from those of tumors predicted to be the least GCN2-dependent (*SI Appendix*, Fig. S9*B*). Clinically, patients with tumors predicted to be GCN2-dependent received pharmacological therapy quicker than patients with a low dependency signature (*P* = 0.0095), developed new tumors faster (*P* = 0.005), and were more likely to receive both pharmacological therapy (*P* = 0.008) and radiotherapy (*P* = 0.015) for these new tumor events. We then repeated the process for DepMap Central Nervous System (CNS) cell lines and found that a 40-gene signature identified 53 out of 697 TCGA glioblastomas/gliomas (7.6%) as GCN2 dependent (*SI Appendix*, Fig. S10*A*). Similar to melanoma patients, they were more likely to receive adjuvant therapy (*P* = 0.004) and to start both pharmaceutical therapy (*P* < 0.001) and radiotherapy (*P* = 0.05) quicker than those with low dependency signatures. Finally, 7 of 361 (1.9%) TCGA hepatocellular carcinomas highly matched a 58-gene signature for GCN2 dependency (*SI Appendix*, Fig. S10*B*). To identify shared molecular features of predicted GCN2-dependency across different cancers, we compared enriched KEGG pathways and found that 17 were communal (*SI Appendix*, Fig. S11*A*). Of those, “Cytokine-Cytokine Receptor Interaction” stood out as the most highly ranked pathway in skin (*SI Appendix*, Fig. S11*B*) and liver and as the third highest ranked in CNS (Table S13). This is biologically relevant because TGF-β pathway genes significantly contributed to the enrichment, which, together with our RNA-sequencing data on GCN2iB-treated MM cells (Fig. 5*C* and Table S11 and *SI Appendix*, Fig. S6*E*), demonstrates a functional link between TGF-β signaling and GCN2 in several malignancies. Moreover, commonly enriched pathways such as “Protein Digestion and Absorption” and “Nitrogen Metabolism” are composed of genes encoding molecules with diverse roles in amino acid transport and biosynthesis, in line with the primary role of GCN2 as a regulator of amino acid homeostasis. Thus, patients with different cancer types that are predicted to have GCN2-dependent tumors share molecular hallmarks that may facilitate stratification for GCN2-targeting therapeutic approaches.

## Discussion

Here, by applying an integrated and temporal systems-level “multiomics” approach, we delineate the global cellular processes by which cancer cells recover from therapy-induced proteotoxic stress, as occurs in vivo in patients treated with PIs (20, 21, 24). Our extended and synchronous profiling of mRNA and protein expression, metabolite levels, and mitochondrial function reveals a layered chart of the intricate and surprisingly protracted mechanisms that are triggered by a brief burst of proteasome inhibition (*SI Appendix*, Fig. S12). We find that the resolution of initial injuries by early stress responses is accompanied by the staggered emergence of new challenges and further corrective measures, resulting in sustained waves of biological processes. The temporal patterns and functional connections of these mechanisms (Fig. 1 *D*–*F*) support a model in which at least some of the challenges that arise in recovering cells are directly linked to the mechanisms of stress resolution. As such, the cellular vulnerabilities that are coupled with the recovery process represent dynamic trade-offs that are distinct from other forms of therapeutically exploited vulnerabilities such as synthetic lethality (48), collateral lethality (49), or drug-induced synthetic lethality that is based on persistent phenotypic changes (50).

The enhanced dependency of recovering cells on GCN2 likely represents one such example. Although levels of some amino acids, such as glutamine and aspartate, dropped to below baseline in the early aftermath of proteasome inhibition, they reached their nadir in later stages of recovery. Moreover, ISR activation and thus dependency on GCN2 signaling became particularly apparent during recovery. The restoration of protein synthesis in recovering cells is a likely explanation for the increased demand for amino acids during stress resolution, and GCN2 blockade in that context is predicted to render the finely tuned attenuation of protein synthesis by the ISR inadequate, allowing protein synthesis to overshoot cellular capacity. Given that protein synthesis depends on degradation (16), our findings that GCN2 inhibition led to increased proteasome subunit expression in recovering cells is compatible with this notion, which is also supported by transcriptome analyses that show enrichment of protein synthesis pathways during recovery (*SI Appendix*, Fig. S2 *B* and *C*). Future studies should therefore address the question if inadequately controlled protein synthesis, which has previously been linked to increased cell death in response to perturbations of the ubiquitin-proteasome system (51–54), is indeed a major mechanism by which GCN2 inhibition perturbs recovery from PIs. Our observations indicate that the role of GCN2 goes beyond regulating the availability of amino acids as protein building blocks. As such, the profound effects of GCN2 inhibition on lipidic metabolites in recovering cells hint at a central role in energy metabolism and are broadly in accord with previously observed functional links between GCN2 and lipid homeostasis (55). While our findings identify GCN2 as a prototypic recovery-associated vulnerability in PI-treated MM cells, they also suggest that a clinically relevant proportion of solid cancers could be amenable to GCN2 inhibition without prior PI treatment, that these cancers may be identifiable by gene-expression signatures, and that they share molecular characteristics linked to key functions of GCN2. While further studies will need to refine the molecular features that define a cancer as GCN2 dependent, our data can form the basis for a drug target discovery pipeline to identify stress-independent targets in subsets of cancer types by means of routine transcriptome analyses.

With a view to a potential clinical application of GCN2 inhibitors, it is worth noting that genetic GCN2 depletion, or its systemic inhibition, is largely well tolerated in murine systems, unless mice receive diets that lack essential amino acids (43, 56, 57). In contrast, inhibition of another eIF2-α kinase, PERK (*EIF2AK3*), has shown promising antitumor effects but is linked to considerable toxicity in mice (58, 59). Further downstream, mitigation of the ISR with Integrated Stress Response Inhibitor (ISRIB), a compound that antagonizes translational reprogramming caused by eIF2-α phosphorylation, has been shown to prevent breast cancer cells from attaining stem-cell–like properties that are required for disease progression (60). Moreover, ISRIB perturbs proteostasis and triggers cytotoxic effects in prostate cancer cells (61). In conjunction with our findings, these studies and others on ER stress (62) highlight the importance of processes linked to eIF2-α in regulating cancer cell fate.

Tumor-promoting roles of GCN2 and its potential as an anticancer drug target have been reported before (43, 63–66), but its relation to proteasome inhibition and role in MM has remained largely undetermined (67, 68). While proteasome inhibition has been shown to trigger lethal amino acid scarcity in yeast, mammalian cells, and flies (15, 51), amino acid depletion has not been a widely accepted mechanism of action of PIs in MM, possibly because it becomes most apparent only when cells begin to recover from proteasome inhibition. Our findings suggest that the ability of MM cells to trigger a GCN2-dependent AAR may contribute to PI resistance, which has been linked to a variety of mechanisms (14, 69). Intriguingly, PI resistance has also been linked to the suppression of 19S proteasome subunits (70–72), and our observation that the expression of several 19S subunit mRNAs dropped below baseline levels in recovering cells could indicate a first step toward resistance development via this mechanism or the persistence of cells with lower 19S subunit expression before treatment. Our finding that GCN2 inhibition markedly increased expression levels of 19S subunits in recovering cells tentatively suggests that GCN2 inhibition could counter this therapeutically unwanted 19S suppression.

Reduced expression of 19S subunits has also been linked to altered mitochondrial energy metabolism as a cause of PI resistance. Induced suppression of the 19S subunit PSMD2 reduces the acute PI-induced drop in OXPHOS, thereby promoting proteotoxic stress tolerance and PI resistance (73). Our findings that mitochondrial respiration was even more suppressed during recovery than during acute stress raises the question whether this state triggers increased or decreased mitochondrial vulnerability. This is particularly relevant in comparison with acutely stressed cells, in which the combination of increased mitochondrial gene expression with suppressed protein levels (Fig. 3 *A*–*D*) suggests a considerable level of mitochondrial stress. Our observations on how mitochondrial stressors affected viability and ATF4 transcript levels are to some extent compatible with a higher level of mitochondrial vulnerability in recovering cells than in acutely stressed cells or unstressed cells, and the effects of metformin on the cellular metabolome we observed support this interpretation. However, future studies need to define the apparently complex role of mitochondria in the resolution of PI stress in more detail if any therapeutic benefit is to be derived. In this respect, it is worth noting that metformin has been linked to reduced progression of the myeloma precursor condition, monoclonal gammopathy of undetermined significance, to overt myeloma (74). The profound metabolic perturbations triggered by metformin in cells recovering from PI treatment can provide the basis for further investigations into combination therapies to suppress myeloma progression.

One of the most striking metabolic changes in the wake of a brief burst of proteasome inhibition that we observed is that glucose consumption and intracellular abundance are reduced even more in cells that are recovering than in acutely stressed cells. These changes are accompanied by a decrease in expression of the glucose transporter, GLUT1, and up-regulated expression of TXNIP, a major suppressor of glucose uptake, in recovering cells (Fig. 2*G*). While the precise mechanisms of action underlying these dynamic changes remain to be determined, it seems plausible that the increased generation of lactate contributes to the up-regulation of TXNIP and suppression of glucose uptake during recovery (30, 31). Intriguingly, TXNIP expression has also been linked to mitochondrial function (75, 76) and is enhanced in response to amino acid depletion (77), suggesting it could be a major metabolic signaling node during PI-stress resolution. Despite activation of the ISR and increased expression of amino acid transporters, cells also failed to recover glutamine levels during recovery. It therefore remains to be established if the scarcity of two of the most important sources of energy and carbon, glucose and glutamine, somehow provides an advantage to recovering cells or is a surprisingly well-tolerated bystander effect. It will also be intriguing to investigate in more detail why the reduction in mitochondrial respiration in recovering cells is so protracted. Our findings indicate that cells recovering from acute PI-induced stress enhance fatty acid catabolism and increase cellular energy generation via β-oxidation (*SI Appendix*, Fig. S12). This metabolic shift could, at least partly, be linked to the increase in proliferation following cell-cycle arrest during acute stress. A similar metabolic state, which is characterized by minimal glycolysis and high dependency on fatty acid oxidation, has been described in rapidly cycling germinal-center B cells (78). Preclinical observations suggest that the reliance of some cancers on fatty acids to generate energy may be exploited therapeutically by means of pharmacological or dietary interventions (79, 80). It is therefore tempting to speculate that such approaches could also be applied in the context of PIs in MM patients, particularly as our observations on the effects of metformin and GCN2 inhibition also link fatty acid metabolism to stress recovery in PI-treated MM cells.

In summary, our work demonstrates that temporal multiomics approaches can reveal metabolic vulnerabilities tied to cellular recovery from chemotherapy, paving the way for new routes to optimize cancer therapies.

## Materials and Methods

A detailed description of all materials and methods used in this study (cell culture and reagents, cell viability assays, quantitative real-time PCR, immunoblotting, RNA-sequencing, TMT labeling proteomics, metabolomics, Seahorse analysis, biomathematic modeling and clustering, statistical analyses, bioinformatic analyses, availability of datasets and code, supplementary references) is available in the online *SI Appendix*. Human mesenchymal stromal cell (hMSC) samples were deidentified prior to use in this study and obtained from the Imperial College Healthcare Tissue Bank (ICHTB, Human Tissue Authority license 12275). ICHTB is approved by the UK National Research Ethics Service to release human material for research (12/WA/0196). Bone marrow aspirates were obtained from healthy pediatric stem-cell donors, and written informed consent for the use of hMSC for research was obtained from the donors’ parents.

## Supplementary Material

Supplementary File

Supplementary File

Supplementary File

Supplementary File

Supplementary File

Supplementary File

Supplementary File

Supplementary File

Supplementary File

Supplementary File

Supplementary File

Supplementary File

Supplementary File

Supplementary File

Supplementary File

## Data Availability

RNA-sequencing, proteomics, metabolomics, and code data have been deposited in Zenodo (https://zenodo.org/record/4010524) and are accessible.
