# Supplementary material for: Systems level profiling of chemotherapy-induced stress resolution in cancer cells reveals druggable trade-offs
Source: Proc Natl Acad Sci U S A. 2021 Apr 21;118(17):e2018229118. doi: 10.1073/pnas.2018229118 (PMC8092411; doi:10.1073/pnas.2018229118)
Supplement: Supplementary File [file pnas.2018229118.sapp.pdf]

PNAS
www.pnas.org

Supplementary Information for:

Systems level profiling of chemotherapy-induced stress resolution in cancer cells reveals druggable trade-offs

Paula Saavedra-Garcia[1,2], Monica Roman-Trufero[1,2,15], Hibah A Al-Sadah[1,2,15], Kevin Blighe[3], Elena Lopez-Jimenez[1,2], Marilena Christoforou[1,2], Lucy Penfold[1,4], Daria Capece[5], Xiaobei Xiong[1,2], Yirun Miao[1,2], Katarzyna Parzych[1], Valentina Caputo[2], Alexandros P Siskos[6], Vesela Encheva[7], Zijing Liu[8,9,10], Denise Thiel[8], Martin F Kaiser[11], Paolo Piazza[12], Aristeidis Chaidos[2], Anastasios Karadimitris[2], Guido Franzoso[5], Ambrosius P Snijders[7], Hector C Keun[6], Diego A Oyarzún [13,14], Mauricio Barahona[8] and Holger W Auner[1,2,]✉

[1]Cancer Cell Protein Metabolism, Department of Immunology and Inflammation, Imperial College London, London, UK. [2]The Hugh and Josseline Langmuir Centre for Myeloma Research, Imperial College London, London, UK. [3]Clinical Bioinformatics Research, London, UK. [4]Cellular Stress, MRC London Institute of Medical Sciences, London, UK. [5]Centre for Molecular Immunology and Inflammation, Department of Immunology and Inflammation, Imperial College London, London, UK. [6]Department of Surgery and Cancer, Imperial College London, London, UK. [7]Proteomics Platform, The Francis Crick Institute, London, UK. [8]Department of Mathematics, Imperial College London, London, UK. [9]Department of Brain Sciences, Imperial College London, London, UK. [10]UK Dementia Research Institute at Imperial College, London, UK. [11]Myeloma Molecular Therapy, The Institute of Cancer Research, Sutton, UK. [12]Imperial BRC Genomics Facility, Department of Metabolism, Digestion and Reproduction, Imperial College London, London, UK. [13]School of Informatics, The University of Edinburgh, Edinburgh, UK. [14]School of Biological Sciences, The University of Edinburgh, Edinburgh, UK.

[15]these authors contributed equally to this work.

✉ Corresponding author: Holger W Auner. E-mail: holger.auner04@imperial.ac.uk

This file includes:

- Figures S1-S13 (page 2-20)
- Materials and Methods (page 21-30)
- Supplementary References linked to Materials and Methods (page 31)

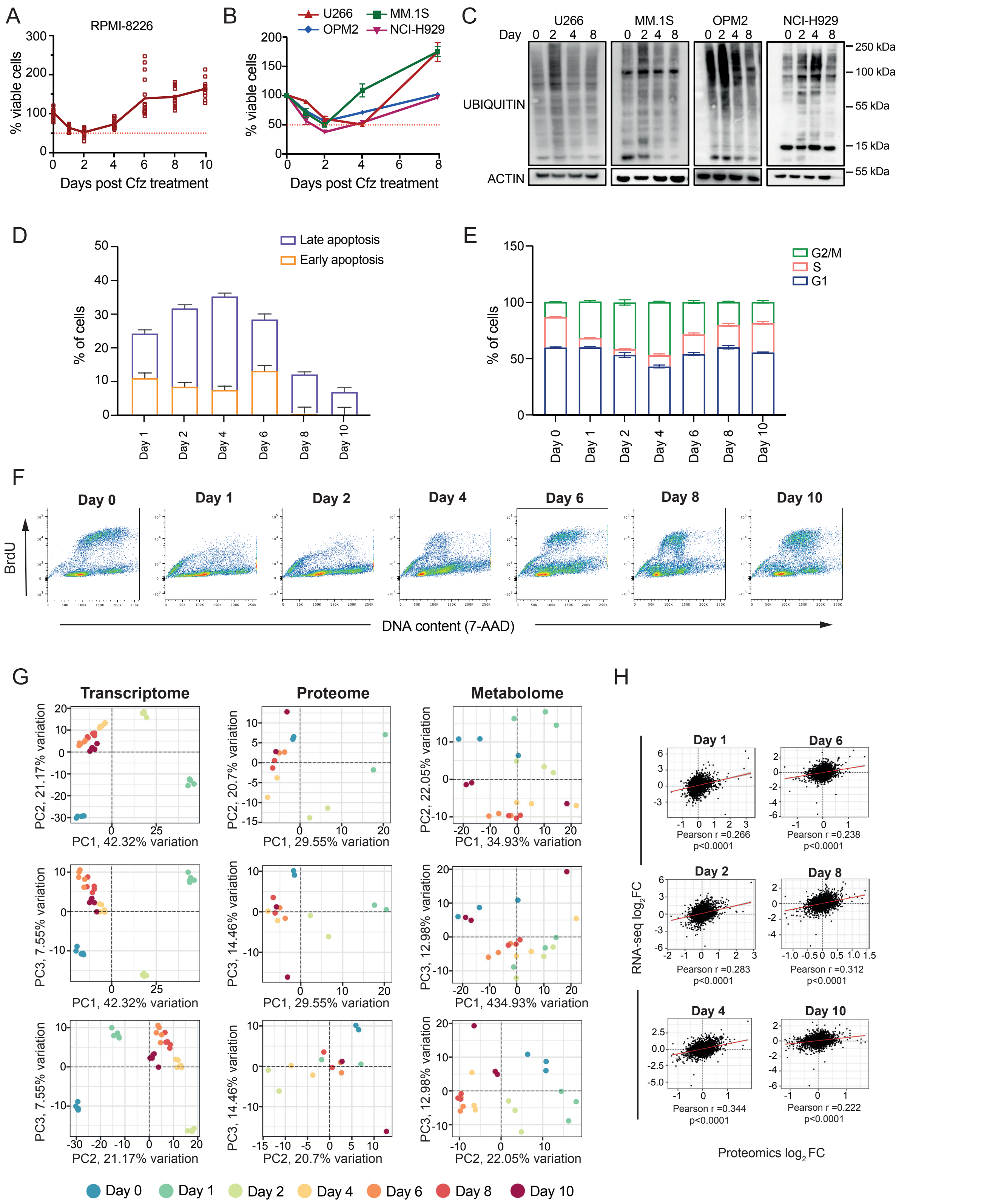

Figure S1. Characterisation of myeloma cell recovery from proteasome inhibition.
A) Percentage of viable cells determined by Guava flow cytometry (n = 16). *B*) Percentage of viable cells of four myeloma cell lines determined by Trypan Blue exclusion (mean ± SEM, n = 3). *C)* Immunoblot analysis of ubiquitinated protein levels in whole cell extracts (representative blot of n = 3). *B*) and *C*)

Carfilzomib (Cfz) doses used for each cell line were MM.1S, 150 nM; OPM2, 150 nM; U266, 750 nM and NCI-H929, 100 nM. *D*) Increase in apoptotic RPMI-8226 cells relative to day 0 as determined by flow cytometry based on AnnexinV-FITC and PI positivity (mean ± SEM, n = 6). *E*, *F*) Cell cycle analysis of RPMI-8226 as determined by flow cytometric analysis after staining with anti-BrdU and 7-AAD. Data are shown in *E)* are mean ± SEM (n = 6), plots shown in *F)* are representative. *G*) Unsupervised principal component (PC) analysis bi-plots of PC1, PC2 and PC3. *H*) Correlation analyses of log_2FC comparisons for all mRNAs and proteins matched by Entrez ID. Red line denotes a linear regression fit with 95% confidence intervals. Captions show Pearson correlation values (r) and correlation test-derived p-values.

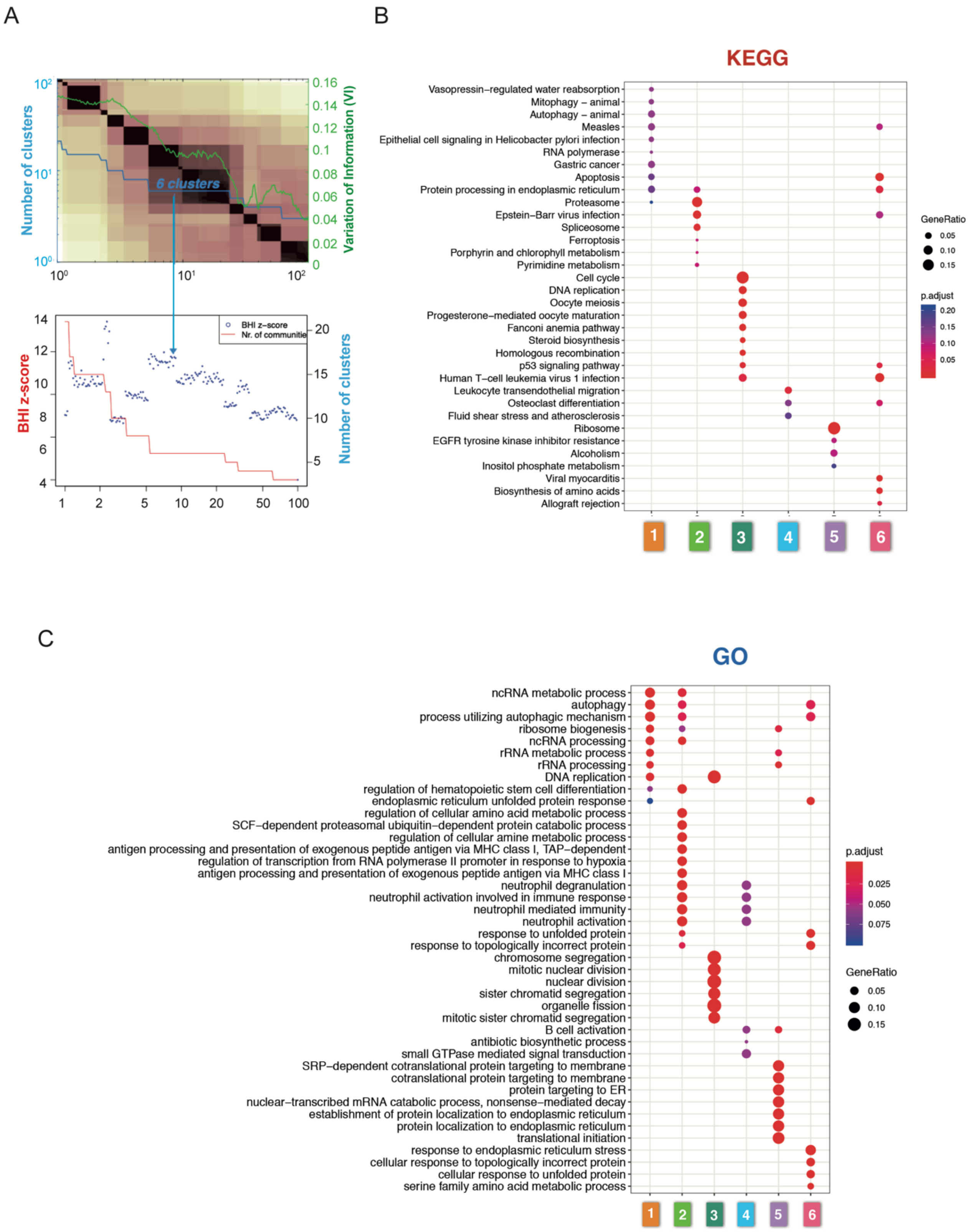

Figure S2. Transcript clustering by Biological Homogeneity Index.
A) Top: Clustering results using Markov Stability on the gene-to-gene similarity network. Blue and green lines show the number of clusters and the variation of information (VI) for a given Markov time. The Markov time specifies a desired resolution of the graph clustering. The heatmap shows the VI between

different clusterings at different Markov times. Blocks of dark areas mean that clusterings are similar and indicate a robust clustering. The same number of clustering across a long Markov time is also a signal of a robust clustering. Bottom: *Biological Homogeneity Index (*BHI) z-scores of the clusterings at different Markov times. The scores indicate how much biological information in the Gene Ontology (GO) is captured by the clustering. From the results shown in both panels, the optimal number of clusters to be applied was determined to be 6. *B*) and *C*) Enrichment of KEGG (*B*) and GO (*C*) terms in the 6 transcriptional clusters.

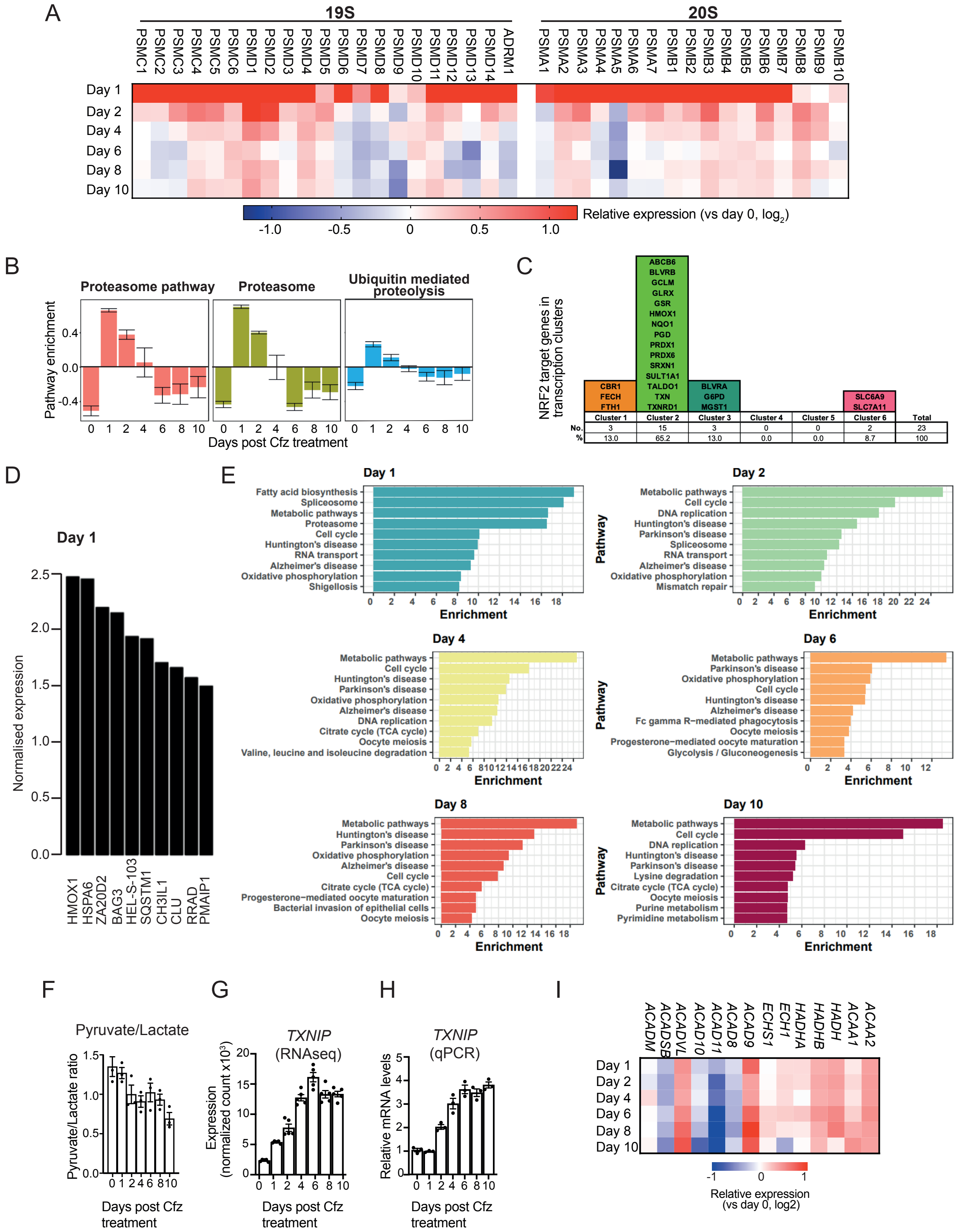

Figure S3. Proteasome inhibitor-induced metabolic pathway changes in RPMI-8226 cells.
A) Heatmap representing relative levels of 19S and 20S proteasome subunit transcripts determined by RNA sequencing (data shown are the mean of five independent experiments relative to day 0). *B*) GSVA of the indicated pathways from independent databases ("Proteasome pathway", Biocarta; "Proteasome", KEGG; "Ubiquitin mediated proteolysis", KEGG). *C*) Distribution of NRF2 target genes in

the BHI-defined transcript clusters. *D*) Ten most highly expressed proteins on day 1 after Cfz treatment as determined by TMT-labelling and shown as mean-normalised expression value. *E*) Cross-validated KEGG pathway enrichment for transcripts with significantly altered expression (Benjamini-Hochberg $Q \leq 0.05$) for each day 1 to 10 comparison to day 0. Enrichment analysis was performed using KEGG profile. Top 10 pathways that pass adjusted $P < 0.001$ are shown for each comparison as negative $\log_{10}$ adjusted p-value. *F*) Pyruvate/lactate ratio in RPMI-8226 cells as determined by LS-MS (mean ± SEM, n = 3). *G*) Expression levels of *TXNIP* measured by RNA-seq (mean ± SEM, n = 5). *H*) Expression levels of *TXNIP* measured by qRT-PCR (mean ± SEM, n = 3 technical replicates). *I*) Relative mRNA levels of β-oxidation enzymes determined by RNA sequencing (data shown as mean of five independent experiments relative to day 0).

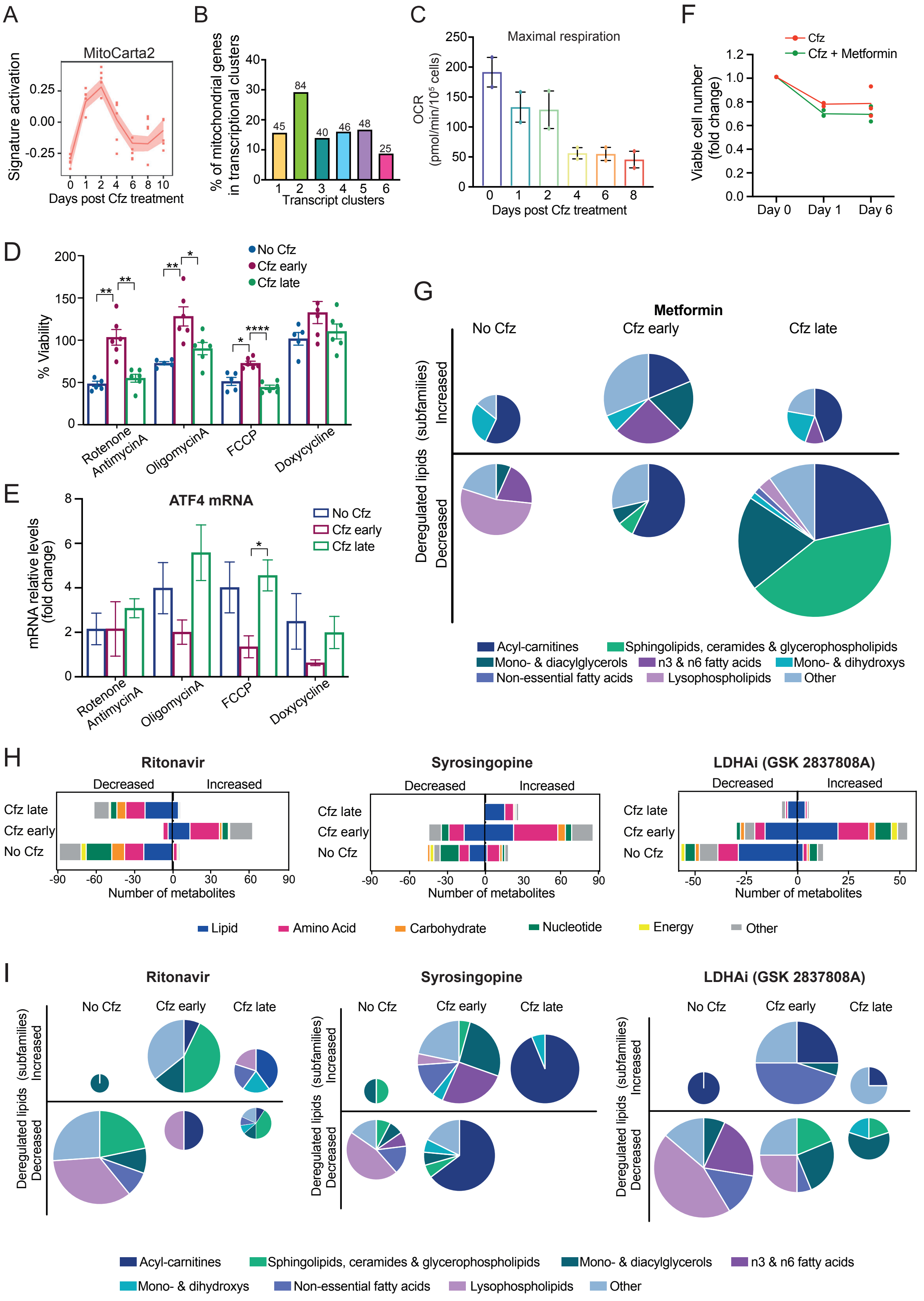

Figure S4. Mitochondrial alterations in proteasome inhibitor-treated RPMI-2886 cells.

A) MitoCarta2.0 signature enrichment determined via GSVA. *B*) Percentage of mitochondrial (MitoCarta2.0) genes present in each of the BHI-transcript clusters. *C*) Maximal oxygen consumption rate (OCR; mean ± SEM, n = 2 independent experiments with 5 technical replicates each) as determined by

Seahorse analysis. *D*) Effect of mitochondrial stressors on the viability of RPMI-8226 cells. Cells were treated with rotenone/antimycin A (1 μM/1 μM), oligomycin A (1 μM), FCCP (10 μM) or doxycycline (5 μg/ml) for 24 h on day 0 (no Cfz), day 1 (Cfz early) or day 5 (Cfz late) and viability was determined by CellTiter 96® Aqueous Non-Radioactive Cell Proliferation Assay (MTS) on days 1, 2 or 6 (mean ± SEM, p-value calculated by two-way ANOVA, Tukey's test for multiple comparison: ****, < 0.0001; **, < 0.01, * < 0.05; n = 6). *E*) mRNA levels of ATF4 in RPMI-8226 cells treated as described in *E*) and determined by qRT-PCR (mean ± SEM, p-value calculated by two-way ANOVA, Tukey's test for multiple comparison: * < 0.05; n = 6). *F*) Viable number of RPMI-8226 cells treated with metformin (1 mM) early (day 1, 24 h) or late (day 6, 24 h) after Cfz treatment, determined by Trypan Blue exclusion (n = 3). *G*) Distribution of significantly deregulated metabolites in lipidic subfamilies in control (no Cfz), or Cfz-treated RPMI-8226 cells (early, day 1; late, day 6; two-way ANOVA, significance threshold < 5% FDR for multiple comparisons). *H*) Number of significantly deregulated metabolites following treatment with ritonavir (1 μM, 24 h), syrosingopine (1 μM, 24 h), or the LDHA inhibitor GSK2837808 (1 μM, 24 h) in control (no Cfz), or Cfz-treated RPMI-8226 cells (early, day 1; late, day 6; two-way ANOVA, 5 % FDR for multiple comparisons). *I*) Distribution of significantly altered metabolites in lipidic subfamilies in RPMI-8226 cells treated as in *H*).

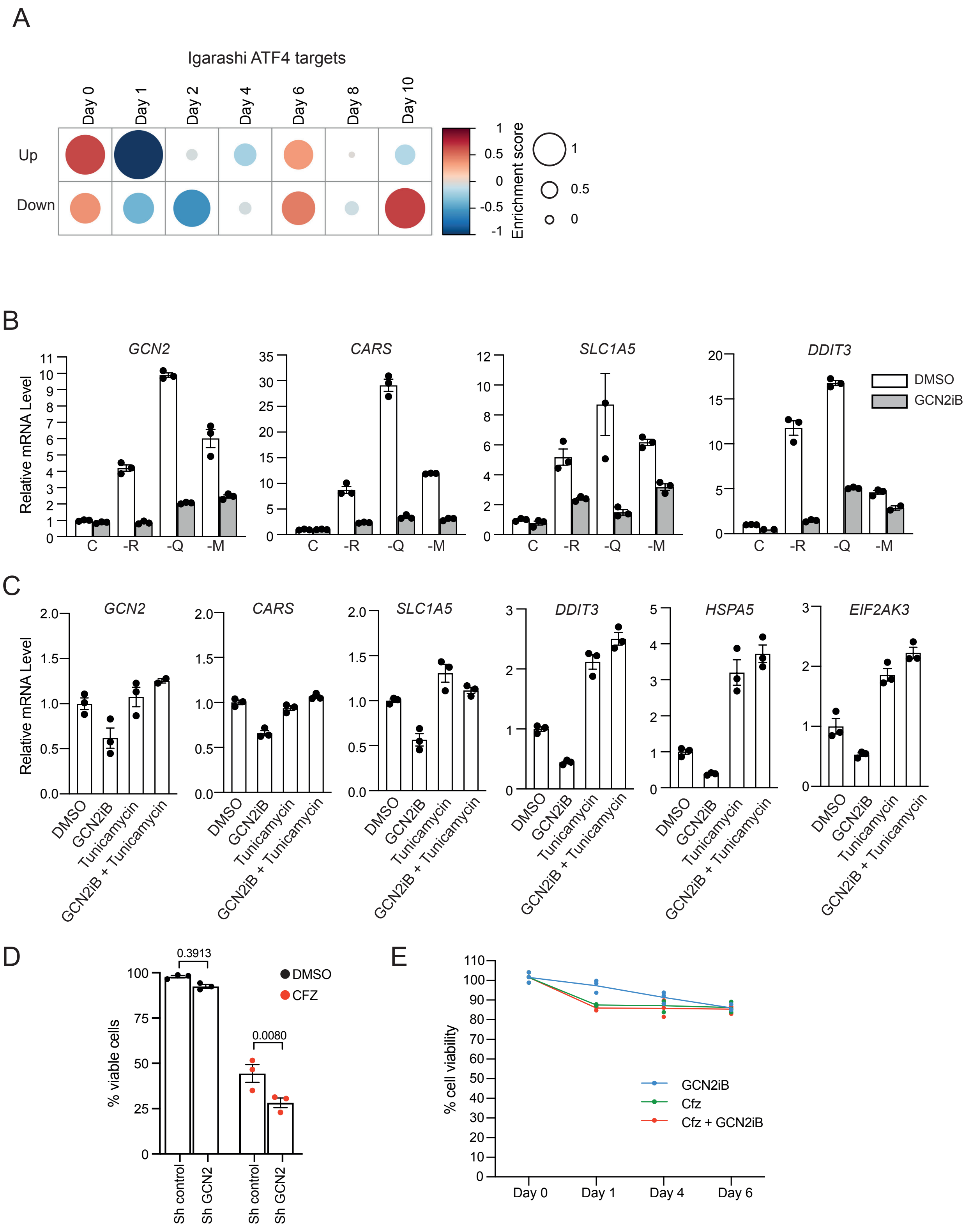

Figure S5. Amino acid response and GCN2 inhibition in RPMI-8226 cells

A) Signature enrichment for *Igarashi ATF4 targets* determined via GSVA. For visualisation, the enrichment score range was scaled to be between -1 (blue) and +1 (red), with white representing 0. The size of the circles is also representative of this scaling, with 0 being the smallest and -1 / +1 being the largest. *B*) Expression levels of GCN2-ATF4 target genes in RPMI-8226 cells grown in conditions of amino

acid depletion, with or without GCN2 inhibition, for 24 h (C, complete; R, arginine; Q, glutamine; M, methionine; GCN2iB, 1 µM). Relative mRNA levels were determined by qRT-PCR (mean ± SEM, n = 3 technical replicates). *C*) Effect of GCN2 inhibition on tunicamycin (5 µg/mL, 24 h)-induced mRNA expression changes. Relative mRNA levels were determined by qRT-PCR (mean ± SEM, n = 3 technical replicates). *Note that GCN2iB largely or completely abrogated the induction of key mRNAs (SLC1A5, CARS, DDIT3, EIF2AK4) by depletion of glutamine, arginine, or methionine from cell culture medium. However, GCN2iB had no or a minimal effect on mRNA upregulation triggered by tunicamycin, which triggers an ATF4-dependent unfolded protein response by activating PERK. Adding to published data (Nakamura et al, Proc Natl Acad Sci USA 115, E7776-E7785 (2018)), the findings demonstrate that GCN2iB is an appropriate pharmacological tool to inhibit amino acid depletion-driven GCN2 signalling.* *D*) A549 cells stably expressing control or GCN2 shRNA were treated with Cfz (750 nM) for 1 h and viability was analysed after staining with AnnexinV and PI by flow cytometry on day 4 (mean ± SEM, two-way ANOVA and Sidak's test for multiple comparisons, n = 3). *E*) Percentage of viable bone marrow mesenchymal stromal cells treated with GCN2iB, after a 1 h pulse of Cfz (250 nM). hMSCs were treated with GCN2iB for 6 days, and viability was determined by RealTime-Glo™ MT cell viability assay (n = 3 technical replicates).

A

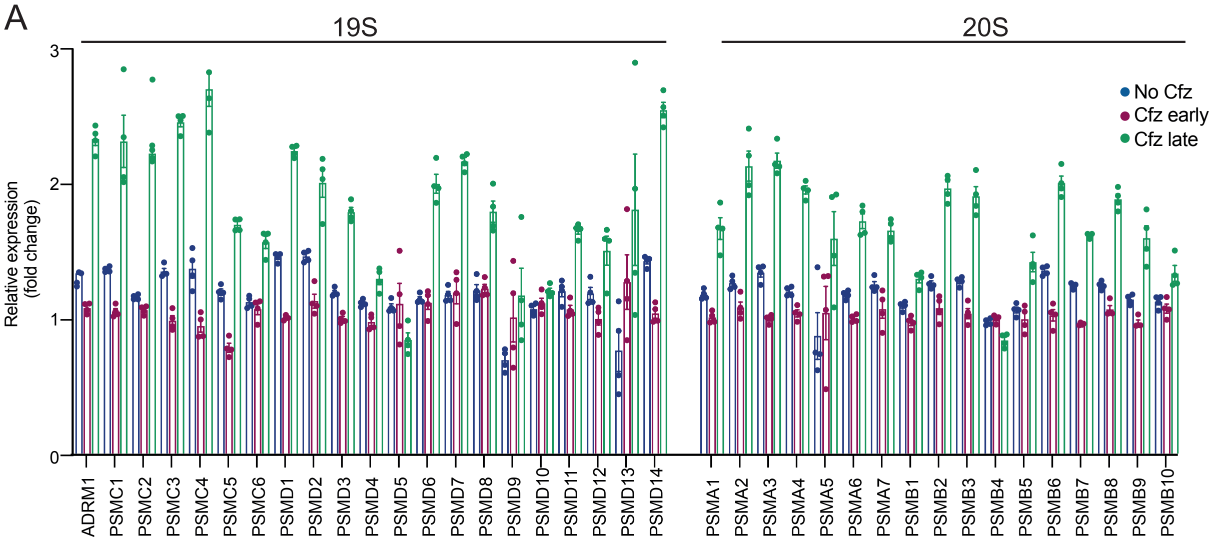

B

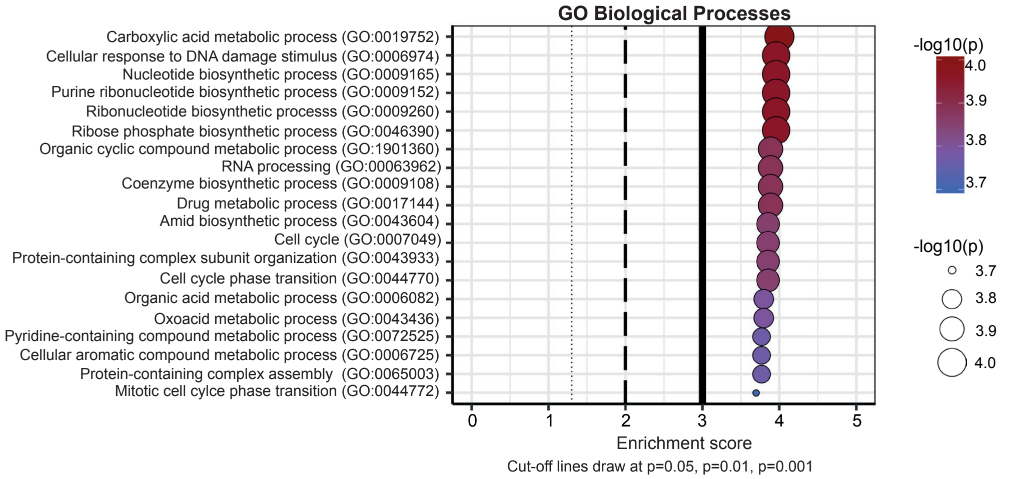

C

	Number of genes	
GO term	Down	Up
Carboxilic acid metabolic process	25	35
Cellular response to DNA damage	10	38
Nucleotide biosynthetic process	4	5
Purine ribonucleotide biosynthetic process	4	2
Organic cyclic compound metabolic process	105	137
RNA processing	11	31

D

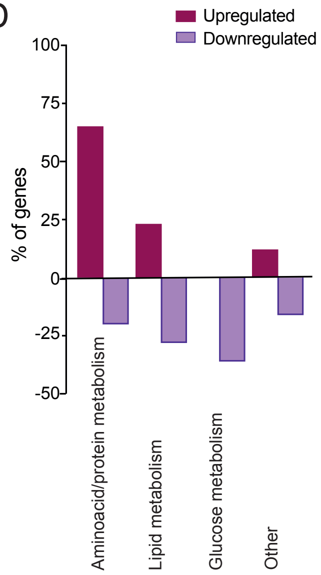

E

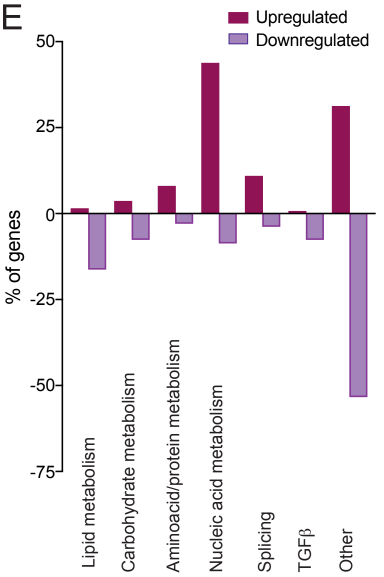

Figure S6. Effect of GCN2iB on the transcriptome of RPMI-2886 cells recovering from proteasome inhibition.

A) Relative expression levels of 19S and 20S proteasome subunits, quantified by RNA sequencing (mean ± SEM, n = 4) in RPMI-8226 cells treated with GCN2iB (1 μM, 48 h) in the absence of Cfz, or early (day 2)

or late (day 4) after a Cfz pulse. *B*) GO biological process term enrichment for differentially expressed genes in RPMI-8226 cells treated with GCN2iB (1 μM, 48 h) in the absence of Cfz and early (day 2) or late (day 4) after a Cfz pulse. Only top 20 enriched terms are shown. Cut-off lines drawn at equivalent of *P* = 0.05 (dotted), *P* = 0.01 (dashed), *P* = 0.001 (solid). *C*) Number of deregulated genes in the 6 most significantly enriched GO Biological Process terms that did not show substantial overlap with other highly ranked terms. *D*) Functional subcategorisation of the 60 deregulated genes included in the GO term "Carboxylic acid metabolic process" (GO: 0019752), *E*) Functional subcategorisation of the deregulated genes included in the GO term "Organic cyclic compound metabolic process" (GO: 1901360).

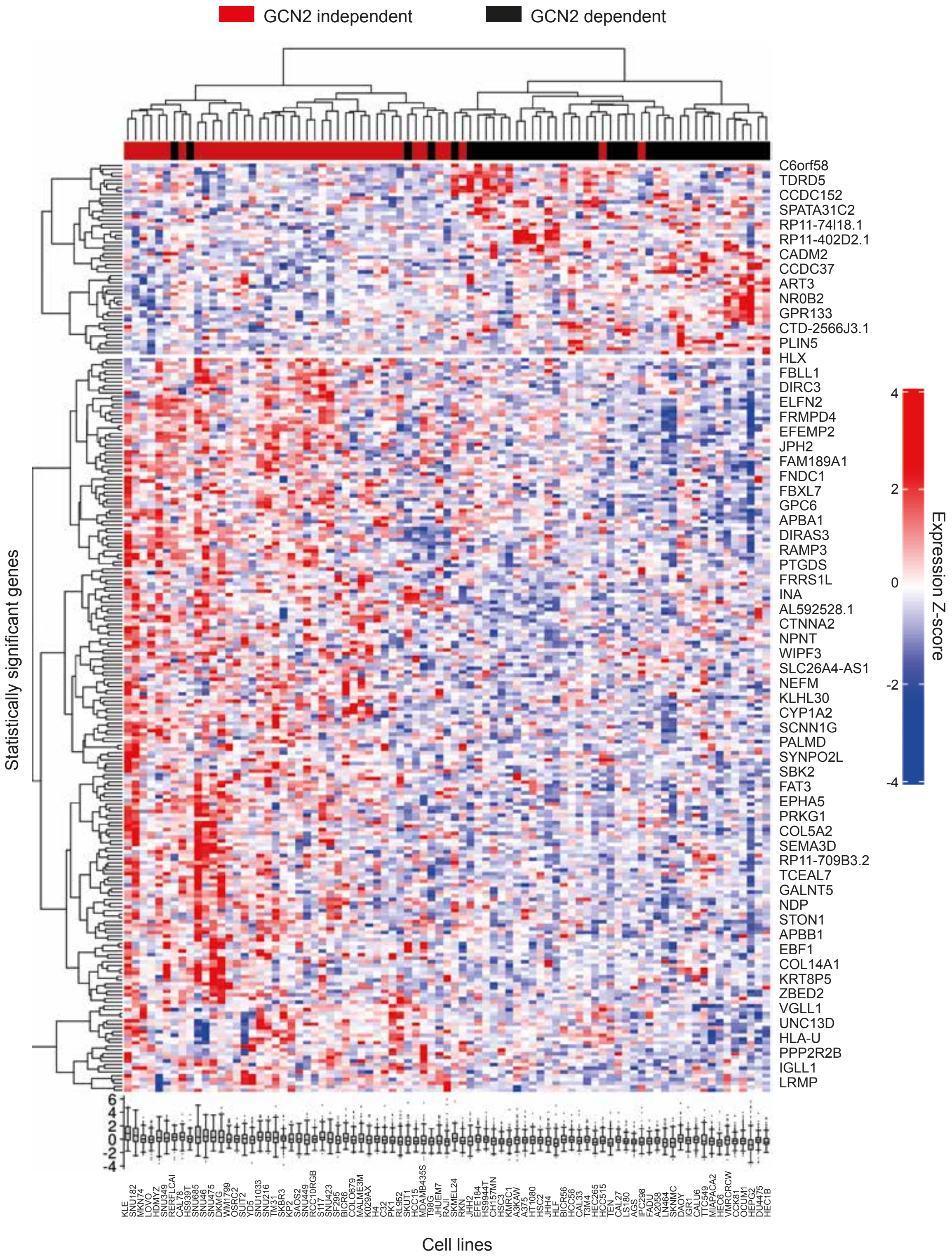

Figure S7. Differential gene expression in GCN2-dependent and GCN2-independent cancer cell lines. Heatmap showing transcriptome differences between GCN2-dependent and -independent CCLE cell lines, based on DepMap CERES scores for GCN2 (*EIF2AK4*) dependency. Differential expression analysis was conducted with DESeq2 with "tissue" as covariate. Normalised counts were transformed via variance stabilisation.

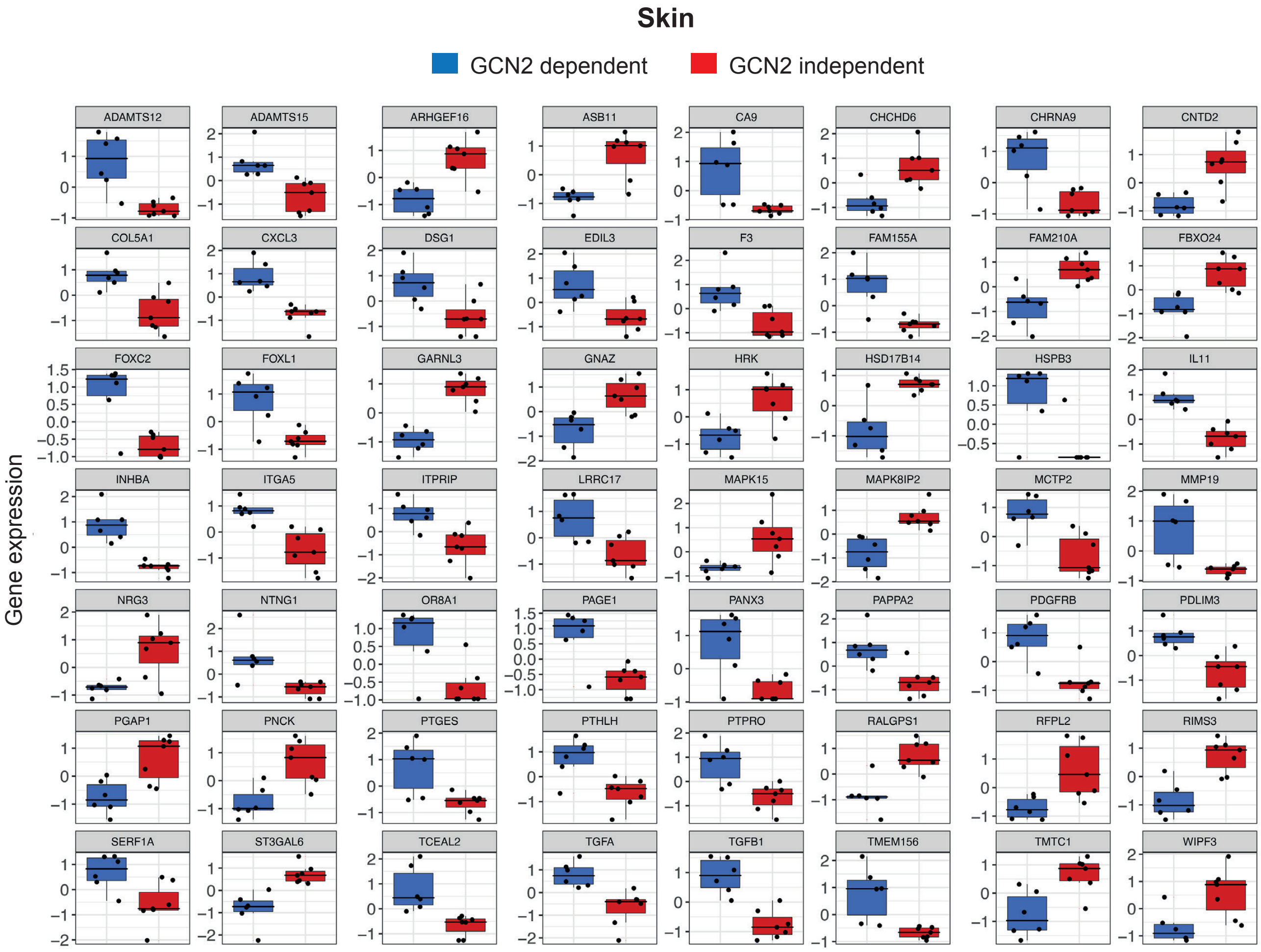

Figure S8. GCN2-dependency signature genes for skin cancer.
Signature genes were identified by 10-fold cross-validated elastic net penalised regression. Box plots show relative mRNA expression in GCN2-dependent/independent cell lines.

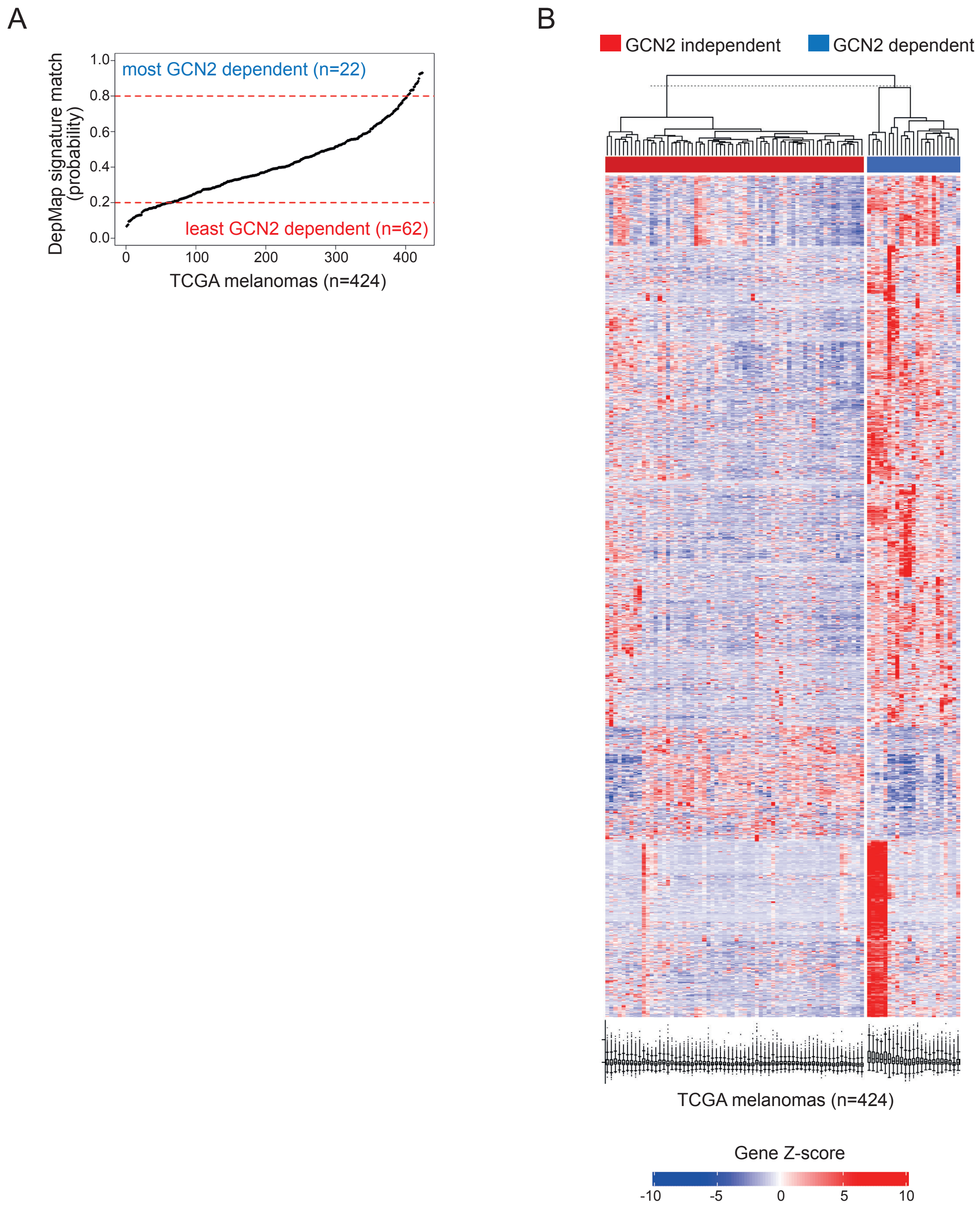

Figure S9. GCN2 dependency in melanoma. *A*) Predicted GCN2-dependency in melanomas based on probabilities of transcriptome matches with a DepMap derived transcriptome signature (cut-offs at 80% and 20% indicate the most and least dependent tumours, respectively). *B*) Heatmap showing transcriptome differences between TCGA melanomas predicted to be the most and least GCN2 dependent as in (*A*).

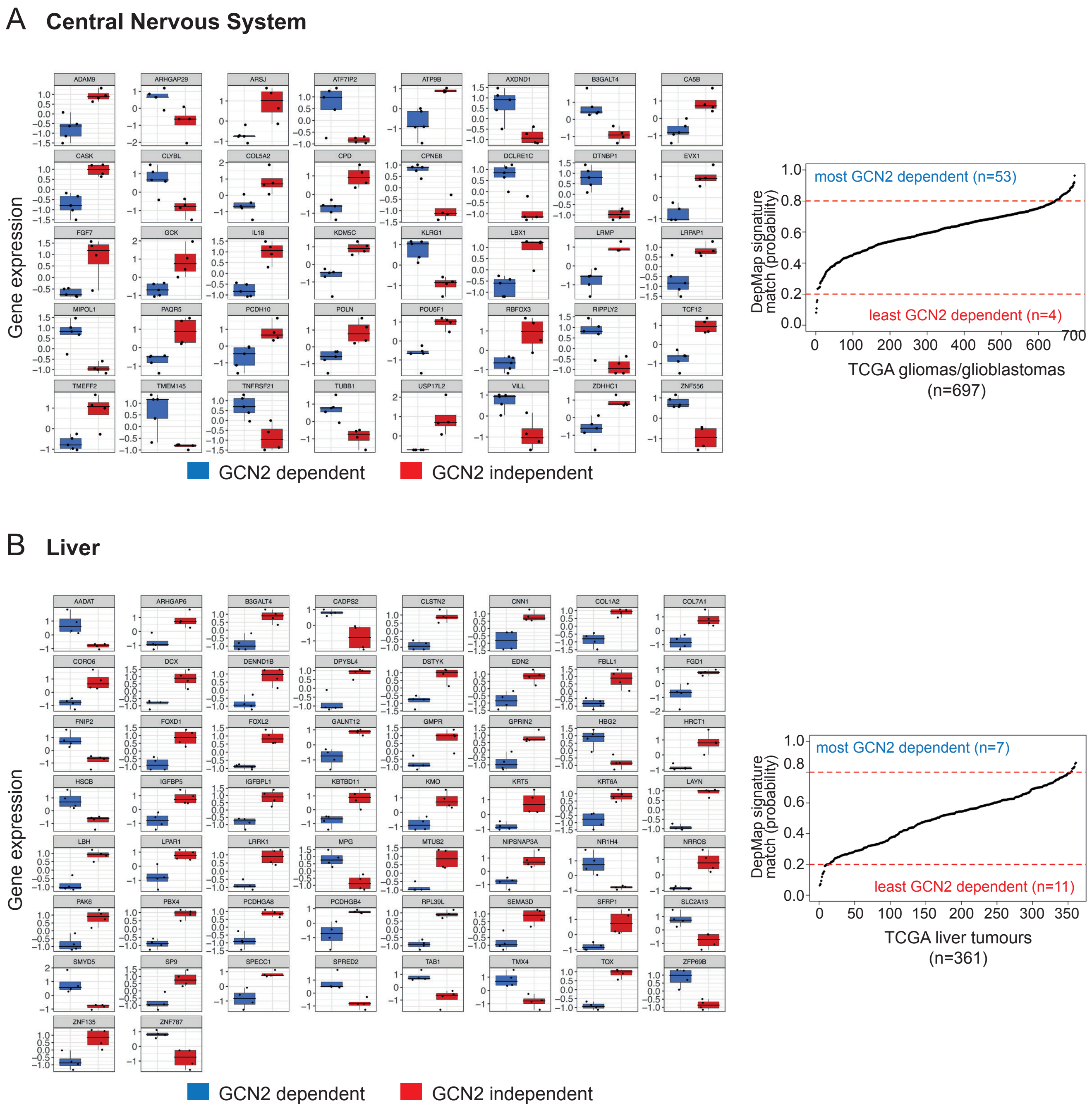

Figure S10. Central nervous system and liver cancer GCN2 dependency signature genes.
A) Left panel, Central Nervous System cancer GCN2-dependency signature genes. Right panel, elastic regression indicating the probability of TCGA glioblastomas to match a DepMap-derived GCN2 dependency signature. *B*) Left panel, liver cancer GCN2-dependency signature genes. Right panel, elastic regression indicating the probability of TCGA liver tumours to match a DepMap-derived GCN2 dependency signature. Signature genes in *A*) and *B*) were identified via 10-fold cross-validated elastic net penalised regression.

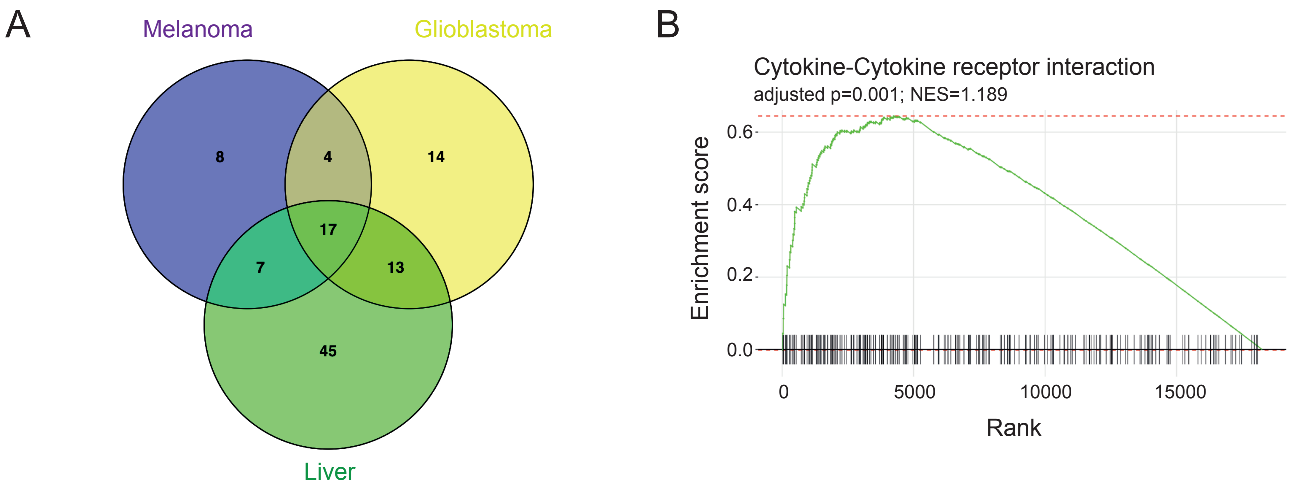

Figure S11. Gene expression features of GCN2-dependent solid tumours. *A*) Venn diagram showing the number of shared and unique enriched KEGG pathways in TCGA melanoma, glioblastoma and liver tumours with high GCN2 dependency signatures. *B*) Gene set enrichment analysis of the KEGG Cytokine-Cytokine Receptor Interaction pathway in TCGA melanomas predicted to be GCN2-dependent, compared to GCN2-independent tumours.

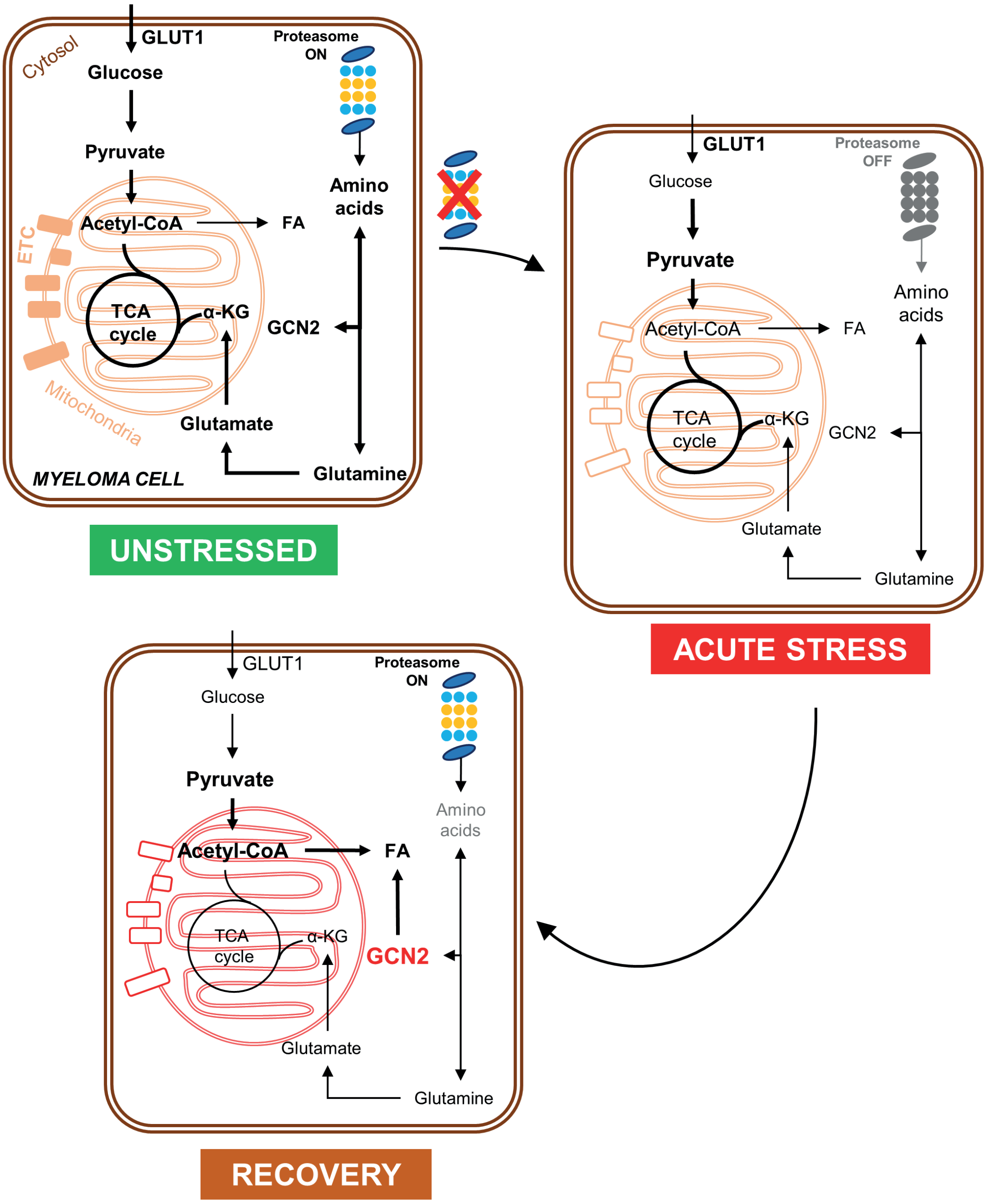

Figure S12. Summary schematic of proteotoxic stress recovery in myeloma cells.
Proteasome inhibition triggers acute proteotoxic stress, which entails a drop in intracellular glucose levels and increased pyruvate availability compatible with heightened glycolytic activity. Tricarboxylic acid (TCA) cycle intermediates and mitochondrial proteins including electron transport chain (ETC) components decrease, while reduced protein degradation leads to amino acid shortages including glutamine scarcity. During stress recovery, glucose uptake becomes yet more suppressed in parallel with reduced GLUT1 expression. Persistent amino acid scarcity triggers GCN2 signalling to regulate fatty acid (FA) metabolism, revealing cellular dependency on GCN2, while mitochondrial respiration and ETC proteins remain suppressed.

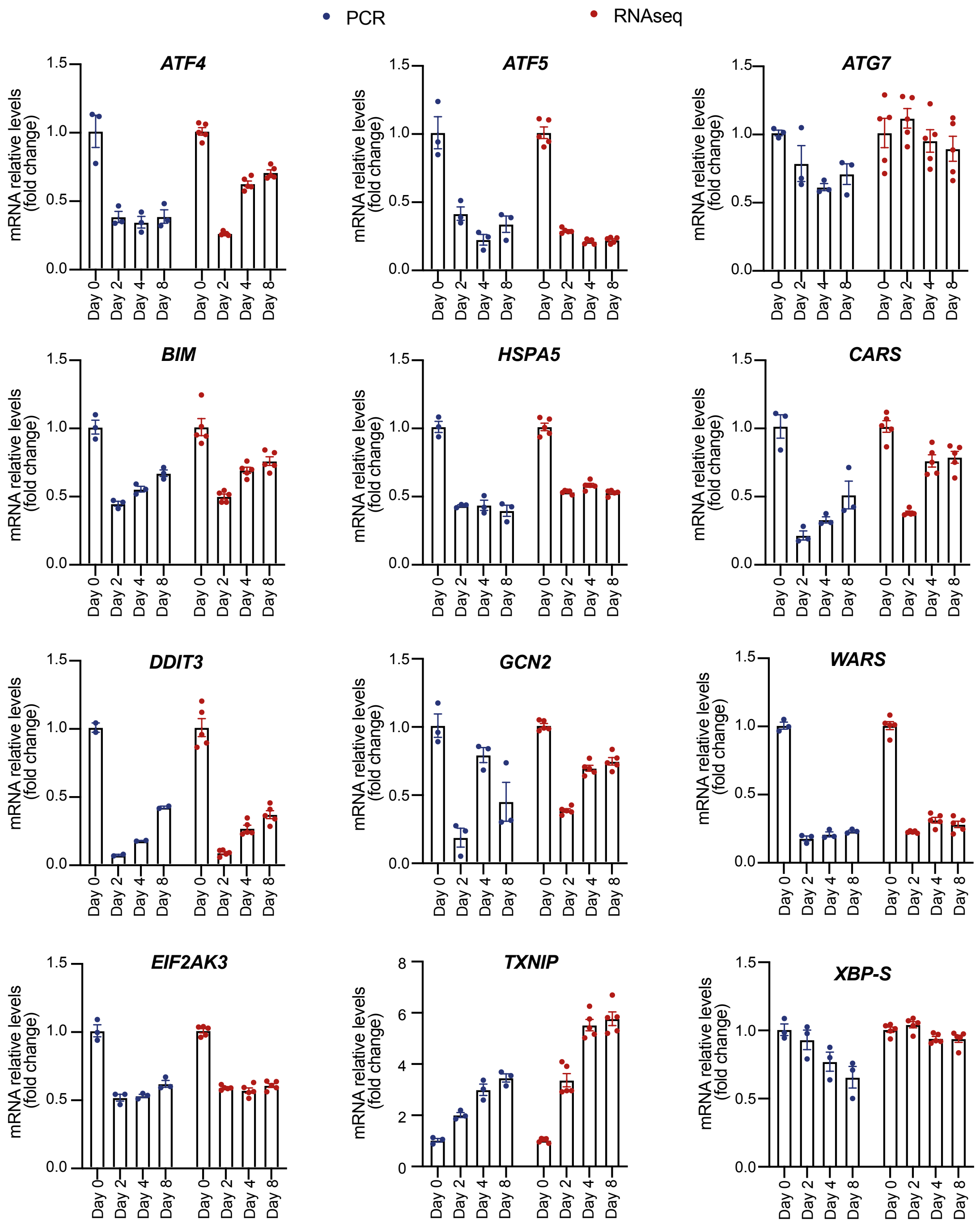

Figure S13. RNA sequencing validation by qRT-PCR.
qRT-PCR validation of gene expression data obtained by RNA sequencing (mean ± SEM; qRT-PCR, n = 3 technical replicates of one randomly selected sample; RNA-seq, n = 5) in a panel of functionally relevant genes.

MATERIALS AND METHODS

Cell culture and reagents.

RPMI-8226 cells were purchased from the European Collection of Authenticated Cell Cultures (ECACC). MM.1S, NCI-H929 and OPM2 cells were from DSMZ-German Collection of Microorganism and Cell Cultures GmbH, and U266 from the American Type Culture Collection (ATCC). Cells were maintained in RPMI-1640 medium (R8758, Sigma-Aldrich) supplemented with 20% foetal bovine serum (FBS, 10500-064, Gibco) and 1% penicillin plus streptomycin solution (Sigma-Aldrich) and kept at a density of 5 x 10^5 to 2 x 10^6 cells/mL. A549 lung adenocarcinoma cells stably expressing shRNA against GCN2 or a control sequence have been described before (1). A549 cells were maintained in DMEM medium (D5796, Sigma-Aldrich) supplemented with 10% FBS (Gibco) and 1% penicillin plus streptomycin solution. Human mesenchymal stromal cells (hMSC) and the ethical procedures regulating their use have been described before (2). hMSC samples were de-identified prior to use in this study and obtained from the Imperial College Healthcare Tissue Bank (ICHTB, HTA license 12275). ICHTB is supported by the National Institute for Health Research Biomedical Research Centre based at Imperial College Healthcare NHS Trust and Imperial College London. ICHTB is approved by the UK National Research Ethics Service to release human material for research (12/WA/0196). hMSC were maintained in MEM medium (11095-080, Gibco) supplemented with 10% FBS and 1% penicillin plus streptomycin solution. All cells were cultured in a humidified atmosphere of 5% CO_2 at 37 °C. Cells were incubated with Carfilzomib (Amgen), GCN2iB (a gift from Justin Bower, The Beatson Institute for Cancer Research, Glasgow, UK), GSK2837808A (5189, Tocris), ritonavir, syrosingopine, antimycin A, rotenone, FCCP, oligomycin A, or doxycycline (SML0491, SML1908, SML0491, SML1908, A8674, R8875, C2920, 75351 and D9891 Sigma Aldrich) as indicated.

Cellular recovery from proteasome inhibition.

RPMI-8226 myeloma cells were expanded to a total of 1.6 x 10^7 cells in 175 cm^2 tissue culture flasks and split into 16 subcultures on day -2. On day 0, cell density was adjusted to 1 x 10^6 cells/mL with fresh medium, and cells were allowed to equilibrate. Carfilzomib (750 nM) was then added to cultures for 1 h. Cells were then washed with PBS and cultured in fresh medium without carfilzomib. Medium was replaced every 48 h and cell density adjusted to 1x10^6 cells/mL on days 6 and 8. Samples for RNA sequencing (n = 5 subcultures), liquid chromatography-tandem mass spectroscopy (n = 3), tandem mass tag labelling proteomics (n = 2), nuclear magnetic resonance spectroscopy of cell culture supernatants (n = 3), quantitative gene expression analysis by qRT-PCR, immunoblotting, and assessment of viability by Trypan Blue exclusion (n = 4) and Guava® easyCyte™ quantification (n = 16) were collected prior to carfilzomib treatment on day 0, after 24 h (day 1), and immediately before changing culture medium on days 2, 4, 6, 8, and 10. Recovery experiments with other cell lines were performed concordantly, with samples collected as indicated. For recovery experiments including GCN2iB, cell density was adjusted to 1x10^6 cells/mL in fresh medium on days 2 and 4.

Cell viability assays.

Cells were mixed 1:1 (volume) with Trypan Blue (93595, Sigma-Aldrich) and then counted using a haematocytometer. In addition, 10 µL of cell culture was mixed with 190 µL ViaCount assay reagent (4000-0040, Merck-Millipore) and incubated for 5 min at RT. Viability data were acquired on a Guava® easyCyte™ instrument (Merck-Millipore) using GuavaSoft 3.1.1 software. Cell viability was also assessed by CellTiter 96® Aqueous Non-Radioactive Cell Proliferation Assay (MTS) (G5430, Promega) according to the manufacturer's instructions in a FLUOstar Omega microplate reader (BMG Labtech). Viable cells were also quantified by flow cytometric analysis using Dead Cell Apoptosis Kit with Annexin V Alexa

Fluor™ 488 & Propidium Iodide (PI) (V13241, Thermo Fisher) according to the manufacturer instruction, using a BD LSRFortessa™ (BD Biosciences). Cell cycle was analysed using FITC BrdU Flow Kit (559619, BD Pharmingen™). RPMI-8226 cells were incubated for 2 h with 10 μM BrdU. Cells were stained for BrdU and 7-ADD according to the manufacturer's instructions, and analysed using a BD LSRFortessa™ (BD Biosciences). Analysis was performed using FlowJo (BD Pharmingen).

Quantitative real-time polymerase chain reaction (qRT–PCR).

Total RNA was extracted with the ReliaPrep™ RNA Miniprep Systems (Z6012, Promega), cDNA was synthesized by GoScript Reverse Transcription System (A5001, Promega). qRT-PCR was performed using PowerUp SYBR Green PCR Master Mix (A25742, Thermo Fisher Scientific) and analysed by StepOne™ Software v2.3 (Applied Biosystems, Thermo Fisher Scientific). mRNA transcript levels were quantified using the standard curve method, GAPDH was used as an internal control for normalization. Gene-specific primers are listed in Supplementary Table 14.

Protein extraction and immunoblot analysis.

Whole cell extracts were prepared by lysing cells in RIPA lysis buffer (89900, Thermo Fisher Scientific), supplemented with protease with phosphatase inhibitors (05 892 970 001 and 04 906 845 001 respectively, Sigma-Aldrich), on ice for 15 min. Lysates were centrifuged at 13000 × g for 10 min at 4 °C and the supernatant was collected and stored at -80 °C. Protein concentration was determined using the Pierce BCA protein assay kit (23225, Thermo Fisher Scientific). Electrophoresis and immunoblot analyses were performed using NuPAGE protein analysis system (Thermo Fisher Scientific). Membranes were blocked with 5% skimmed milk in TBS plus 0.1% TWEEN® 20 (TBS-T) for 1 h at RT and incubated with primary antibodies (diluted 1: 1,000) overnight at 4 °C. Membranes were then washed with TBS-T and incubated with the appropriate secondary antibody (1:2500). Bands were visualised using enhanced chemiluminescence (ECL) reagents (RPN2232, Amersham Pharmacia) with a ChemiDoc image system with Image Lab software, version 5.2 (Bio Rad). The antibodies used in this study are: actin (A3853, Sigma Aldrich), NDUFB8 (total OXPHOS, ab110413, Abcam), ubiquitin (3933), TXNIP (14715), GLUT1 (12939), GLUT4 (2213), Mouse Anti-Rabbit Immunoglobulins/HRP (7076P2) and Rabbit Anti-Goat Immunoglobulins/HRP (7074S) from Cell Signalling Technology. PageRuler Plus Prestained Protein Ladder (26619, Thermo Fisher Scientific) was used as a molecular weight marker.

RNA-sequencing.

Total RNA from 2 x 10^6 cells per sample was extracted with the ReliaPrep™ RNA Miniprep Systems kit (Promega). RNA samples were assessed for quantity and integrity using a Tapestation High Sensitivity RNA ScreenTape (Agilent), and 100 ng of total RNA were used for sequencing. Libraries were constructed using the NEBNext® Ultra™ II Directional RNA Library Prep Kit for Illumina (E7760L, New England Biolabs) according to manufacturer recommendations. Briefly, after fragmentation to approximately 180 nucleotides fragment size, the samples were converted to double stranded DNA and ligated to Illumina adapters. The second strand cDNA was then degraded by the UNG enzyme providing directional information to the library. Each library was uniquely indexed by PCR. Libraries obtained from each sample were quantified and quality-checked on a Tapestation using the High Sensitivity D1000 ScreenTape system prior to pooling. A HiSeq4000 was used to generate 75 paired-end sequencing reads according to manufacturer recommendations. qRT-PCR of a panel of 12 representative genes was performed to confirm key expression data (Supplementary Fig. S13).

Tandem mass tag (TMT) labelling proteomics.

Sample preparation and digestion.

Whole cell extracts were prepared from 100 µg per sample. To reduce cysteine residues, tris (2-carboxyethyl) phosphine (TCEP, C4706-2G, Sigma-Aldrich) was added to a final concentration to 10 mM and the samples were incubated at 55 °C for 1 h. Subsequently, the reduced proteins were alkylated with 20 mM iodoacetamide (T7408-100ML, Sigma-Aldrich) for 30 min, protected from light, at RT. Protein precipitation was performed with 1:6 volume of pre-chilled (-20 °C) acetone overnight at -20 °C. After overnight incubation, lysates were centrifuged at 8000 × g, for 10 min, at 4 °C. The supernatant was discarded, and the pellets were air-dried for 10 min to remove residual acetone and then dissolved in 100 µL of 100 mM TEAB. Trypsin (Trypsin Gold, Mass Spectrometry Grade, Promega) was added at a 1:40 (trypsin: protein) ratio and digestion was allowed to proceed at 37 °C for 18 h.

TMT Labelling and Sample Fractionation.

After in-solution digestion the samples were labelled with 0.2 mg of TMT isobaric label reagents reconstituted in anhydrous acetonitrile (Thermo Fisher Scientific). Peptide samples containing 50 µg of protein were labelled with the corresponding TMT 10-plex reagent for 1 h at RT. The reactions were quenched using hydroxylamine added to a final concentration of 0.3% (v/v) for 30 min. TMT-labelled peptides were combined and fractionated using Pierce High pH Reversed-Phase Peptide Fractionation Kit (Thermo Fisher Scientific) following the manufacturer's instructions. All fractions (nine fractions in each set) were dried by vacuum centrifugation and stored at -80 °C until mass spectrometric analysis.

LC-MS/MS analysis.

Peptide mixtures from TMT 10-plex labelled samples were chromatographically resolved on an EASY-spray PepMap RSLC C18 column (2 µm, 100Å, 75 µm × 50 cm ID) using an Ultimate 3000 RSLCnano system (Thermo Fisher Scientific) over a 180 min gradient at 40 °C. The peptides were separated using linear gradient of 2-45% solvent B over 145 min. The column was washed with 95% B for 15 min and equilibrated with 2% B for the rest of the acquisition. Solvent A was 0.1% FA and 5% DMSO in HPLC-grade water, and solvent B was 0.1% FA and 5% DMSO in 80% acetonitrile and the flow rate was 250 nL/min. All spectra were acquired using an Orbitrap Fusion Lumos Tribrid mass spectrometer (Thermo Fisher Scientific) and Xcalibur 4.2.28.14 (Thermo Fisher Scientific) was used to control data acquisition. The instrument was operated in data dependent acquisition mode with top scan speed set at 3 s. MS1 spectra were acquired in the Orbitrap at a resolution of 120000 and an ion target of 4E5. The MS2 precursors were isolated using the quadrupole (0.7 Th window) and analysed in the Orbitrap at a resolution of 60000, with an AGC target of 1E5 and a max injection time of 118 ms. Precursors were fragmented by HCD at a normalized collision energy (NCE) of 38%.

Data processing and analysis.

Orbitrap.RAW files were analysed by MaxQuant (version 1.6.0.13), using Andromeda for peptide search. MS2 spectra were searched against the Uniprot database specifying *Homo sapiens* (Human) taxonomy (Proteome ID: UP000005640, Organism ID: 9606, number of entries: 21039). A list of 247 common laboratory contaminants provided by MaxQuant was also added to the database. All searches were performed using "Reporter ion MS2" with "10-plex TMT" as isobaric labels. For the search the enzyme specificity was set to trypsin with maximum of two missed cleavages. The precursor mass tolerance was set to 20 ppm for the first search (used for mass re-calibration) and to 4.5 ppm for the main search.

Carbamidomethylation of cysteines was specified as fixed modification, oxidized methionines and N-terminal protein acetylation were searched as variable modifications. Reporter mass tolerance was set to 0.003 Da. TMT signals were corrected for isotope impurities based on the manufacturer's instructions. The dataset was filtered on posterior error probability to achieve 1% FDR on peptide and protein level.

LC-MS metabolomics.

Sample Preparation.

10 x 10^6 live cells as determined by Trypan Blue exclusion were used per sample. Briefly, samples were prepared using the automated MicroLab STAR® system. Several recovery standards were added prior to the first step in the extraction process for quality control purposes. To remove protein, dissociate small molecules bound to protein or trapped in the precipitated protein matrix, and to recover chemically diverse metabolites, proteins were precipitated with methanol under vigorous shaking for 2 min, followed by centrifugation. The resulting extract was divided into five fractions: two for analysis by two separate reverse phase (RP)/UPLC-MS/MS methods with positive ion mode electrospray ionization (ESI), one for analysis by RP/UPLC-MS/MS with negative ion mode ESI, one for analysis by HILIC/UPLC-MS/MS with negative ion mode ESI, and one sample was reserved for backup. Samples were placed briefly on a TurboVap® (Zymark) to remove the organic solvent. The sample extracts were stored overnight under nitrogen before preparation for analysis.

Ultrahigh Performance Liquid Chromatography-Tandem Mass Spectroscopy (UPLC-MS/MS).

The sample extract was dried and then reconstituted in solvents compatible to each of the four analysis. Each reconstitution solvent contained a series of standards at fixed concentrations to ensure injection and chromatographic consistency. One aliquot was analysed using acidic positive ion conditions (chromatographically optimized for more hydrophilic compounds), and the extract was gradient eluted from a C18 column (Waters UPLC BEH C18 - 2.1 x 100 mm, 1.7 µm) using water and methanol, containing 0.05% perfluoropentanoic acid (PFPA) and 0.1% formic acid (FA). Another aliquot was also analysed using acidic positive ion conditions and the extract was gradient eluted from the same afore mentioned C18 column using methanol, acetonitrile, water, 0.05% PFPA and 0.01% FA and was operated at an overall higher organic content. Another aliquot was analysed using basic negative ion optimized conditions using a separate dedicated C18 column. The basic extracts were gradient eluted from the column using methanol and water, with 6.5 mM ammonium bicarbonate at pH 8. The fourth aliquot was analysed via negative ionization following elution from a HILIC column (Waters UPLC BEH Amide 2.1 x 150 mm, 1.7 µm) using a gradient consisting of water and acetonitrile with 10 mM ammonium formate, pH 10.8. The MS analysis alternated between MS and data-dependent MSn scans using dynamic exclusion. The scan range varied slighted between methods but covered 70-1,000 m/z.

Data Extraction and Compound Identification.

Raw data was extracted, peak-identified and quality control-processed using Metabolon's hardware and software. These systems were built on a web-service platform utilizing Microsoft's .NET technologies, which run on high-performance application servers and fiber-channel storage arrays in clusters to provide active failover and load-balancing. Compounds were identified by comparison to library entries of purified standards or recurrent unknown entities. Metabolon maintains a library based on authenticated standards that contains the retention time/index (RI), mass to charge ratio (m/z), and chromatographic data (including MS/MS spectral data) on all molecules present in the library. Furthermore, biochemical identifications are based on three criteria: retention index within a narrow RI

window of the proposed identification, accurate mass match to the library +/- 10 ppm, and the MS/MS forward and reverse scores between the experimental data and authentic standards. The MS/MS scores are based on a comparison of the ions present in the experimental spectrum to the ions present in the library spectrum. Metabolites were normalised to dsDNA concentration of the samples.

Metabolite consumption and release rate.

High-resolution 1-dimensional 1H NMR spectroscopy was used to profile metabolite consumption and release (3). Frozen culture supernatant samples were thawed at RT and subsequently vortexed. A volume of 550 µL of each sample was transferred into microcentrifuge tubes, 50 µL of DSS buffer (12 mM deuterated 4, 4-dimethyl-4-silapentane-1-sulfonic acid, 0.5 M sodium phosphate, 0.02% sodium azide in D_2O water) were added to each sample before tubes were vortexed and centrifuged at 20000 x g for 1 min. A volume of 550 µL of each sample supernatant was then transferred into 5 mm diameter Wilmad® NMR tubes (Sigma-Aldrich, Missouri, US).

High-resolution 1-dimensional 1H NMR spectroscopy was performed using the 14.1 T Bruker AVANCE 400 MHz spectrometer (Bruker Biospin) at 298 K. NMR spectra were acquired for each sample using a conventional ZGPR solvent pre-saturation method with a single RF pulse, a recycle delay of 4 s, spectral width of 6 402.049 Hz, 32 FIDs and 64000 data points. Data were automatically digitalised, and Fourier transformed before being processed in MATLAB® software (Mathworks) using in-house scripts developed in the Department of Surgery and Cancer at Imperial College London (London, UK). Phase correction, baseline correction, ppm referencing (DSS) and normalisation to the reference peak (DSS) was automatically done before spectral peaks were identified with reference to the Human Metabolome Database (HMDB) and subsequently integrated using in-house scripts in in MATLAB® software (Mathworks). Metabolite consumption and release rate (molar amount per cell and per unit time) was calculated by subtracting the measured concentration (mM) of fresh supernatant (C_{fresh}) to spent supernatant (C_{spent}),normalizing (divided) by the average number of viable cells ($\bar{x}_{cells}$), and by the time difference in hours between the current and the previous time point (Δt),

$$Metabolite\ comsuption\ and\ release\ rate = \frac{C_{spent} - C_{fresh}}{\bar{x}_{cells} * \Delta t}$$

Seahorse analysis.

Oxygen consumption rate (OCR) was determined using a Seahorse XFe96 Analyzer (Agilent) following manufacturer's recommendations. Briefly, Seahorse cartridges (102601-100, Agilent) were hydrated and incubated at 37 °C in a CO_2-free incubator for 24 h before analysis. Seahorse 96-well plates (102601-100, Agilent) were coated with 0.01% poly-lysine solution (4832, Sigma Aldrich). On the day of the analysis, the Seahorse XFe96 analyser was calibrated using the hydrated cartridge. A total of 10^5 live RPMI-8226 cells per well were plated in XF assay medium (102365, Agilent) complemented with 10 mM glucose (G8769, Sigma Aldrich), 1 mM sodium pyruvate (1136007, Gibco) and 2 mM glutamine (25030081, Gibco) and incubated for 1 h at 37 °C without CO_2. OCR was quantified after injection of 1.5 µM oligomycin A, 60 µM 2,4-Dinitrophenol (DNP) (34334, Sigma Aldrich), or 1 µM rotenone/antimycin A. Results were acquired and analysed by the Wave 2.6.1 software (Seahorse bioscience Inc.). Data were normalized to viable cell numbers at the time of analysis as determined by Trypan Blue exclusion, and day 0 to 8 samples were analysed in parallel on one plate.

Gene expression time series modelling and clustering.

RNA-sequencing-derived transcript counts for 18062 genes were regularised and $\log_2$-transformed using DEseq2(4). Low counts were filtered out by applying an expression cut-off at the trough between the two modes of the distribution of the log2-transformed sum of normalized counts. This resulted in 11798 transcripts selected for further analysis. To study the dynamics of the recovery with respect to the expression levels in day 0, we removed the expression data on day 0 (timepoint t_1) from analysis and re-normalised the remaining timepoints by subtracting the mean of the $\log_2$-transformed regularised counts for the 11798 transcripts. Lastly, we filtered for genes that underwent substantial changes in their expression profiles. To this end, we computed the variance:

$$\mathrm{var}(x_i) = \frac{1}{29}\sum_{j=1}^{30}\left(x_i(t_j) - \mu_i\right)^2,$$

with μ_i being the expression level averaged across all timepoints. We applied a cut-off $\mathrm{var}(x_i) > 0.1$, which resulted in 2542 genes selected for further analysis.

We modelled the time series with a combination of Gaussian Process (GP) regression and graph-based clustering (5). For GP regression we employed a squared exponential function as a covariance function and learned the GP hyper-parameters by maximizing the marginal likelihood for all 2542 genes(4). We then used GP likelihood to compute a similarity score between every pair of genes, which were then converted into a gene-to-gene similarity graph. For clustering, we pruned the graph using k-nearest neighbour (kNN) algorithm with $k = 7$, where a gene is connected only to its seven nearest neighbour genes based on the GP similarity. When the kNN graph had disconnected components, we intersected it with the minimum spanning tree of the fully connected graph. The resulting graph was clustered with the Markov Stability (MS) algorithm for community detection(5). MS produces different graph partitions depending on the Markov time, a resolution parameter that allows to examine the graph structure across scales. We determined the number of clusters by three simultaneous criteria: 1) the algorithm identifies the same number of clusters for a wide range of Markov times (blue curve in SI Appendix Fig. S2*A*); 2) the identified clusters are stable, as quantified by the variation of information across Markov times (green curve and heatmap SI Appendix Fig. S2*A*); 3) the clusters are biologically meaningful, as quantified by the biological homogeneity index (BHI) (6). Based on these criteria the filtered genes were clustered into 6 groups (SI Appendix Fig. S2*B,C*). The average gene expression profile for each cluster was computed with GP regression of the genes in each cluster. For enrichment analysis we employed the R package clusterProfiler to identify the Gene Ontology (GO) terms and KEGG pathways that were over-represented by the clusters of genes (7). A p-value cut-off of 0.05 was used to retrieve relevant terms and pathways.

Statistical analysis.

Unless stated otherwise, data show means and standard error of the mean (SEM) from at least three independent experiments. Statistical tests are indicated in the figure legends and were performed using GraphPad Prism 8.4.2 software. Differences among groups were considered statistically significant at $P < 0.05$ unless stated otherwise.

Bioinformatic analyses and datasets.

Datasets and code are available at https://doi.org/10.5281/zenodo.4010524.

RNA-sequencing.

Read count abundances were generated from raw data FASTQ files by pseudo-aligning these to the *Homo sapiens* GENCODE 'comprehensive' reference transcriptome (GRCh38.p12 / release 28) using Kallisto v0.44.0, adjusting for GC content bias and bootstrapping 50x. Bootstrapped transcript isoform-level counts were then imported to R Programming Language v 3.5.0 (R) and summarised to gene level counts (adjusting for gene-length) using the tximport v1.9.8 (8) package in R.

Protein coding genes (total = 19665) were then isolated from the raw counts based on GENCODE biotype keyword 'protein_coding'. A gene with zero counts across all samples was removed (total = 1603). The raw counts of the remaining genes (total = 18062) (as a tximport object) were then converted to a DESeq2 v1.25.8(4) object for normalisation with betaPrior set to FALSE. For downstream analyses, the negative binomial-distributed normalised counts were transformed to regularised log (rlog) and variance-stabilised (vst) expression levels via the rlog and vst functions of DESeq2 in R, with blind set to FALSE.

Differential expression analysis was conducted on the negative binomial-distributed normalised counts with FDR set at 5%. Following differential expression, log_2 fold changes (log_2FC) were shrunk via the lfcshrink function of DESeq2. A gene was defined as differentially expressed if it passed Benjamini-Hochberg $Q \leq 0.05$ and absolute $log_2FC \geq 2$ unless stated otherwise.

Proteomics.

Raw data was processed and normalised using Perseus (9, 10). Log_2FCs were also derived for days 1-10 via comparisons to day 0 ('vs. day 0' comparisons) - sequential comparisons (e.g., day 0 vs day 1; day 1 vs day 2; day 2 vs day 4; et cetera) were also derived ('sequential day' comparisons). limma v3.41.15 (11) was used to perform the differential expression comparisons. Test statistics were modified via the empirical Bayes methods via ebayes.

Metabolomics.

The raw area count metabolite levels were normalised and imputed by Metabolon as follows: 1, each sample was normalised by dsDNA concentration; 2, each metabolite was rescaled to have median = 1; and 3, missing values were imputed with the minimum overall value. These values were then log_2 transformed and converted to the Z-scale for the purposes of all downstream analyses. Differential metabolite analysis was performed using RegParallel (https://github.com/kevinblighe/RegParallel. doi:10.18129/B9.bioc.RegParallel), which fits a linear model independently to each metabolite level, with final nominal p-values adjusted for FDR (Benjamini-Hochberg).

Principal component analysis (PCA).

Principal component analysis was performed using PCAtools (https://github.com/kevinblighe/PCAtools. doi:10.18129/B9.bioc.PCAtools). Bi-plots were generated to compare eigenvectors, i.e., principal components (PCs), 1 to 3. The percent variation contributed by each PC to the total dataset variation was also calculated and illustrated via a scree plot for QC purposes.

Volcano plots.

Volcano plots were generated using the EnhancedVolcano package v1.3.1 (https://github.com/kevinblighe/EnhancedVolcano. doi:10.18129/B9.bioc.EnhancedVolcano). Plots were then plotted together using grid.arrange from the gridExtra v2.3 package.

Clustering and heatmaps.

Supervised (filtered) and unsupervised (unfiltered) clustering were performed using the Heatmap function of the ComplexHeatmap v2.1.0 package (12). Data was transformed to the Z scale (scaled by row/gene) and then clustered via 1 minus Pearson correlation distance (or Euclidean distance) and Ward's linkage. A samples box-and-whisker plot of Z-scores was added to the heatmap bottom. In certain cases, k-means clustering was performed on heatmap data in order to identify *k* clusters, with the variable-to-cluster assignment then being used to split the heatmap and dendrogram into *k* separate entities.

Fold-change comparisons: RNA-seq vs. Proteomics.

For fold-change comparisons, the log_2FC for all RNA-seq and proteomics genes/proteins that matched on Entrez ID were included, irrespective of statistical significance. biomaRt v2.41.7 (13, 14) was used to convert gene symbols to Entrez IDs. Log_2FCs were then extracted for day 0 comparisons for these genes/proteins and then plotted via geom_point from ggplot2. A linear regression fit with 95% confidence intervals was added via geom_smooth and stat_smooth. Pearson correlation values and the p-values from the correlation test were added as captions. Plots were arranged via grid.arrange from the gridExtra package.

GO term and KEGG pathway enrichment.

For GO term enrichment, topGO v2.37.0 was used. Enrichment was made against “BP” (biological processes), “CC” (Cellular Component), and “MF” (Molecular Function) ontology with nodeSize = 5. The “classic” algorithm with the Kolmogorov-Smirnov (K-S) test, which takes the rank of each target gene into consideration, was used to determine statistically significantly enriched terms ($P < 0.001$) - only top 10 terms were chosen.

Pathway enrichment for KEGG was performed using KEGGprofile v1.27.0. Initial enriched pathways were identified via the find_enriched_pathway function, with returned_pvalue = 0.01, returned_adjpvalue = 0.05, and and returned_genenumber = 5, or returned_genenumber = 3 for the GCN2-dependency TCGA signature enrichments due to lesser numbers of genes in each signature. This process was repeated 10x to increase reliability of results, randomly selecting 90% total genes during each bootstrap, and selecting the lowest obtained adjusted p-value where enriched pathways were the same across multiple bootstraps. The final list of pathways was then filtered by adjusted $P < 0.001$. Prior to topGO and KEGGprofile, gene symbols were converted to Entrez IDs via biomaRt, with non-matching symbols being removed. For the GCN2-dependency TCGA signatures, no cross validation was performed, again due to lesser numbers of genes in each signature.

For each comparison, genes passing Benjamini-Hochberg $Q \leq 0.05$ were used. Final plots were drawn via geom_bar from ggplot2, as negative log_{10} of the enrichment p-value.

Gene-set variation analysis.

Unbiased/Unsupervised gene-set variation analysis (GSVA) was performed using GSVA v1.33.1 (15) and using the C2 curated gene sets from Molecular Signatures Database (C2 MsigDB), with parameters min.sz = 5, max.sz = 999999, and abs.ranking = FALSE. Input in each case were rlog expression levels with gene symbols converted to Entrez IDs via biomaRt. Gene symbols that did not match were removed. For comparisons to day 0, limma was used to fit a linear model to the enriched dataset for the purpose of deriving test statistics. Test statistics were modified via the empirical Bayes methods via ebayes. Separately, GSVA enrichments were also made against subsets of C2 MSigDB signatures. These subsets were identified via key terms as indicated in the manuscript. The resulting enrichments were then plot via corrplot v0.84, with enrichment level scaled between -1 and +1.

Mitocarta 2.0 analyses.

Genes/Proteins passing Benjamini-Hochberg Q ≤ 0.05 were included, and these were separated for those that were down- or up-regulated (negative or positive fold-change, respectively), and presence / absence in MitoCarta2.0. A Pearson $\chi 2$ test was performed at each day to gauge the statistical significance of MitoCarta2.0 vs non-MitoCarta2.0 statistically significant genes. ggplot2 was then used to plot count frequencies of these as a stacked bar plot, with each bar colour-coded based on MitoCarta2.0 / non-MitoCarta2.0 genes/proteins.

Gene-set variation enrichments using GSVA were also performed separately against just MitoCarta2.0 known mitochondrial genes, i.e., MitoCarta2.0 was the signature being enriched.

DepMap GCN2 dependency and TCGA.

Raw RNA-seq counts were downloaded from CCLE (https://portals.broadinstitute.org/ccle) as *CCLE_RNAseq_genes_counts_20180929.gct.gz*, with accompanying metadata *Cell_lines_annotations_20181226.txt*. Counts over cell-lines of interest were extracted and then different analyses were conducted using DESeq2:

- Normalisation of all samples followed by differential expression between cell-lines with highest DepMap GCN2 dependency (loCERES) and lowest GCN2 dependency (hiCERES), with inclusion of "tissue" as covariate.
- Separate normalisations and differential expression (as above) for each main tissues of interest (CNS, liver, and skin) separate.

Downstream heatmaps were generated as previously described in these methods.

TCGA HTSeq raw counts for glioblastoma multiforme (GBM), lower grade glioma (LGG), skin cutaneous melanoma (SKCM), liver hepatocellular carcinoma (LIHC), and head & neck squamous cell carcinoma (HNSC) were downloaded from Xena (https://xenabrowser.net/datapages/). Separate sample metadata in biotab format was downloaded from the Genomic Data Commons (GDC) Data Portal (https://portal.gdc.cancer.gov/). Normal samples were filtered out of each dataset based on the TCGA barcode. Further filtering at the raw count stage was performed as follows:

- SKCM, filter exclude *primary_melanoma_known_dx == NO*
- LIHC, filter include *histologic_diagnosis == Hepatocellular Carcinoma*

These were then normalised via DESeq2 - variance-stabilised expression levels were also produced for downstream analyses.

For the construction of tissue-specific predictive models (one each for CNS, liver, and skin) using the CCLE data, we first identified statistically significant genes between loCERES vs. hiCERES at Benjamini-Hochberg Q ≤ 0.05 and $|log_2FC| \geq 1$ for each tissue. Using glmnet (16), we then fit an elastic-net penalised regression model and cross-validated this x10. The best predictors from this model were identified as those whose coefficients were not shrunk to zero based on the 1 standard error rule (lambda.1se). Enriched GO terms and KEGG pathways were identified from these genes as previously described in these methods.

For each tissue specific CCLE model, we then made predictions on each respective TCGA dataset, i.e.: CNS → GBM + LGG; skin → SKCM; liver → LIHC. Prior to model predictions, the variance-stabilised expression levels of each TCGA dataset was standardised by subtracting, per gene, the mean, and then dividing by the standard deviation. Only TCGA samples with predictions at > 80% probability were retained. Where possible, and numbers per predicted class permitted, a differential expression analysis was conducted between the loCERES and hiCERES predicted TCGA samples. A logistic regression analysis was also performed against the TCGA biotab clinical data to discover statistically significant associations.

For gene-set enrichment analysis, we used the fgsea package in R. Enrichment was made against selected KEGG pathway with 100000 permutations. Input genes were ranked based on negative log of the adjusted p-value for the predicted low versus high GCN2-dependency TCGA samples.

SUPPLEMENTARY REFERENCES

1. K. Parzych *et al.*, The coordinated action of VCP/p97 and GCN2 regulates cancer cell metabolism and proteostasis during nutrient limitation. *Oncogene* **38**, 3216-3231 (2019).
2. S. Loaiza *et al.*, An engineered, quantifiable in vitro model for analysing the effect of proteostasis-targeting drugs on tissue physical properties. *Biomaterials* **183**, 102-113 (2018).
3. M. Jain *et al.*, Metabolite profiling identifies a key role for glycine in rapid cancer cell proliferation. *Science* **336**, 1040-1044 (2012).
4. M. I. Love, W. Huber, S. Anders, Moderated estimation of fold change and dispersion for RNA-seq data with DESeq2. *Genome Biol* **15**, 550 (2014).
5. J. C. Delvenne, S. N. Yaliraki, M. Barahona, Stability of graph communities across time scales. *Proc Natl Acad Sci U S A* **107**, 12755-12760 (2010).
6. S. Datta, S. Datta, Methods for evaluating clustering algorithms for gene expression data using a reference set of functional classes. *BMC Bioinformatics* **7**, 397 (2006).
7. G. Yu, L. G. Wang, Y. Han, Q. Y. He, clusterProfiler: an R package for comparing biological themes among gene clusters. *Omics : a journal of integrative biology* **16**, 284-287 (2012).
8. C. Soneson, M. I. Love, M. D. Robinson, Differential analyses for RNA-seq: transcript-level estimates improve gene-level inferences. *F1000Res* **4**, 1521 (2015).
9. S. Tyanova *et al.*, The Perseus computational platform for comprehensive analysis of (prote)omics data. *Nat Methods* **13**, 731-740 (2016).
10. J. Cox, M. Mann, 1D and 2D annotation enrichment: a statistical method integrating quantitative proteomics with complementary high-throughput data. *BMC Bioinformatics* **13 Suppl 16**, S12 (2012).
11. M. E. Ritchie *et al.*, limma powers differential expression analyses for RNA-sequencing and microarray studies. *Nucleic Acids Res* **43**, e47 (2015).
12. Z. Gu, R. Eils, M. Schlesner, Complex heatmaps reveal patterns and correlations in multidimensional genomic data. *Bioinformatics* **32**, 2847-2849 (2016).
13. S. Durinck, P. T. Spellman, E. Birney, W. Huber, Mapping identifiers for the integration of genomic datasets with the R/Bioconductor package biomaRt. *Nat Protoc* **4**, 1184-1191 (2009).
14. S. Durinck *et al.*, BioMart and Bioconductor: a powerful link between biological databases and microarray data analysis. *Bioinformatics* **21**, 3439-3440 (2005).
15. S. Hanzelmann, R. Castelo, J. Guinney, GSVA: gene set variation analysis for microarray and RNA-seq data. *BMC Bioinformatics* **14**, 7 (2013).
16. J. Friedman, T. Hastie, R. Tibshirani, Regularization Paths for Generalized Linear Models via Coordinate Descent. *J Stat Softw* **33**, 1-22 (2010).
